# Investigating AVHs narratives through text analysis: the proposal of Dialogic Science for tackling stigmatization

**DOI:** 10.1186/s40359-024-01936-x

**Published:** 2024-08-10

**Authors:** Davide Bassi, Luisa Orrù, Christian Moro, Davide Salvarani, Gian Piero Turchi

**Affiliations:** 1 Centro Singular de Investigacion en Tecnoloxìas Intelixentes (CiTIUS), University of Santiago de Compostela, Rúa de Jenaro de la Fuente Domínguez, Santiago de Compostela, 15782 Spain; 2 Dipartimento di Filosofia, Sociologia, Pedagogia e Psicologia Applicata (FISPPA), University Of Padua, Via Venezia, 14, Padova, 35131 Italy; 3 Associazione Nazionale “Sentire le Voci”, Via Fratelli Manfredi, 6, Reggio Emilia, 42124 Italy

**Keywords:** Auditory verbal hallucinations, Stigma, Qualitative research, Text analysis, Dialogic Science

## Abstract

**Background:**

Auditory verbal hallucinations (AVHs) are a significant symptom of various psychological conditions, often stigmatized and misunderstood. Moving beyond traditional psychological, psychotherapeutic and psychiatric approaches, recent research shifts focus on understanding AVHs through community perspectives and the resulting stigmatization. This research approach is crucial for better support and understanding of AVHs, however it still suffers from the lack of a rigorous and shared methodology for studying and reducing stigma.

**Methods:**

Our study, part of the Italian “PsicoVoice” project, aims to investigate community discourses on AVHs, in order to observe whether and to what extent they are drivers of stigmatisation processes. Engaging 268 participants with direct (hearers) and indirect (such as relatives and professionals) experiences of AVHs, the research analyzes a corpus of 54,320 instances using MADIT: a text analysis methodology which is both qualitative and quantitative. MADIT allows for an innovative examination of the rhetorical-argumentative structures within narratives, producing an index for measuring the narratives’ practical impact on people’ interactions around AVHs.

**Results:**

The analysis revealed that the overall community discourses are predominantly shaped by absolute and personal belief-driven modalities. This way of conveying sense, even with non-necessarily-judgmental words, contributes to a stigmatizing environment for individuals with AVHs, cementing a static representation dominated by personal opinions and reducing the potential for more nuanced, diverse interactions about AVHs.

**Conclusion:**

The study’s findings underscore the importance of addressing the narrative structures within community discourses. By intervening in these narratives, there is potential to shift towards a less stigmatizing social construction of AVHs. Thus, the article concludes using the results to provide some insights on how to generate these interventions. This approach could significantly impact how communities understand and interact with individuals experiencing AVHs, promoting more inclusive and supportive environments and interventions.

## Introduction

### AVHs’ stigma: the need for an interactions analysis

Auditory verbal hallucinations (henceforth AVHs), commonly known as hearing voices, have a reported prevalence in the general population ranging from 0.6% to 84%, with a median of 13.2% [1]. While often associated with psychiatric disorders, AVHs also occur in psychologically healthy individuals [2, 3]. Different studies suggest that a significant portion of those experiencing AVHs have no diagnosable disorder [4].

Despite its prevalence among non-clinical people, voice hearing is still strongly associated with pathology and mental illness, leading to significant stigma [5]. This stigma can have a detrimental impact on the individual’s experience of their voices and their recovery, as well as potentially influencing the intensity and frequency of the AVHs themselves [6]. Moreover, stigma surrounding hearing voices is particularly prevalent among healthcare professionals and students, contributing to a cycle of negative attitudes and treatment outcomes [7].

The compelling need to address the stigmatization towards AVHs is further underscored by the initiatives of the Hearing Voices Movement (henceforth HVM). One of the key values of this movement is to promote a conceptualization of AVHs as an always possible element in an individual’s biography. This exactly to reduce the stigma surrounding the theme of AVHs and, at the same time, to empower the subjects dealing with it [8].

To tackle stigma, different intervention formats are available. Direct contact with voice hearers has shown to be effective in reducing stigma among healthcare professionals, students, and the general public [5, 7]. However, the effectiveness of other typology of interventions varies, with some, like simulations, potentially increasing stigma [9–11]. Educational interventions aimed at dispelling myths about mental illnesses generally reduces stigma, but biogenetic explanations can foster perceptions of danger [12, 13]. The variety of these results accounts for the necessity to develop more rigorous conceptualizations and measures for stigmatization. This could help in developing more pertinent and precise interventions and, at the same time, more rigorous measures for their effectiveness.

On the research side, stigma has been traditionally studied from an individualistic paradigm (focused on motivation and cognitive processes) or a societal one (focused on economical, political and historical factors) [14]. More recent approaches, instead, have embraced a narrative and discursive point of view on AVHs’ stigma. Following the definition provided by Link and Phelan [15], in fact, stigma is built in situated social interactions. The elements on which these interactions are structured belong to different domains, but they all share a common root: the use of natural language.

The analysis of language interactions proved to be particularly effective for the analysis of stigma, being aptly equipped for investigating the multifaceted nature of this theme [16, 17]. As Stutterheim and Ratcliffe [18] pointed out, focusing on how language is used is particularly appropriate to understand and change stigma, since: (a) it implies participatory research, offering substantial opportunities for meaningful community engagement, which promotes agency and empowerment, and redresses power imbalances [19]; (b) it ensures that future research questions and study designs are informed by the lived experiences of individuals and/or communities with a stigmatized identity or condition, reducing the risk that research findings are driven by (potentially flawed) assumptions on the part of non-community member researchers [20]; (c) it can contributes to an effective stigma reduction, tailoring the intervention objectives and strategies on the specific needs of the people involved [21].

Considering the pivotal role that language analysis can have in studying stigma, in this paper we describe the results of “PsicoVoice”: an Italian national-level research carried out in 2022 and 2023 by the University of Padova (FISPPA Department) and “Associazione Nazionale Sentire le Voci”^1^, an Italian association supporting AVH-related issues. The project collected and analyzed AVHs narratives generated by different roles of the community (for more details see Results section) to promote more inclusive and supportive environments and interventions with respect to AVHs; thus, improve how communities understand and interact with individuals experiencing this phenomenon in order to contribute in tackling the stigma revolving around them.

The rest of the paper proceeds as follows: Methods and materials section - “Methods and materials” deepens the theoretical and methodological references adopted for the analysis of the text, as well the structure of the questionnaire and the characteristics of the sample. Results section - “Results” describes the results for the different research areas of the questionnaire and the different roles. Discussion section - “Discussion” discusses the pragmatic implications that can be anticipated from the analysis of the results. Finally, Conclusion section - “Conclusion” describes some operational suggestions we elaborated both for the study and the contrast of stigma towards AVHs.

## Methods and materials

### Dialogic Science for the processual analysis of stigma

The analysis of the gathered texts was performed referring to Dialogic Science and MADIT (Methodology for the Analysis of Computerized Text Data) [22, 23].

Dialogic Science studies the interactive processes among humans through the use of natural language. Natural language, hence, is considered as a feature of the human species that allows people to interact between them, even when speaking different idioms: in fact, these latter are local shapes of natural language, which subsumes all idioms [24].

In light of this, Dialogic Science shifts the analysis’ focus from the semantic dimension of the narrative to the processual one, i.e. on the ways speakers employ language to construct and negotiate the social construction of reality, shape discursive scenarios and create shared narratives (for additional details on the processual understanding of language see [24]). In other words, how people shape different discursive configurations related to a given topic [25].

These configurations, in turn, are understood as sets of rhetorical-argumentative joints - i.e. parts of the narrative where the way of conveying sense changes (e.g. descriptive, provisional, judgmental, etc.) - which shape the overall reality of sense in a peculiar way [23].

To perform this analysis, Dialogic Science encoded 24 Discursive Repertories (henceforth DRs) [26, 27]. Each DR corresponds to a specific way to organize the elements of the discursive productions that can be employed in discursive interaction (see Appendix-A for the complete list).

Below we exemplifies how the same two contents “auditory hallucinations” and “school difficulties” can be connected according to different rhetorical-argumentative structure (additional examples are provided in the Results section):

- To establish an absolute causal link: “*auditory hallucinations inevitably provoke poor school performance and relationships*” [DR of “Cause”]
- To establish a possibilistic link: “*auditory hallucinations may potentially negatively influence school performance and relationships*” [DR of “Possibility”]
- To provide a description: “*during school years, we observed students with auditory experiencing poor school performance and strained relationships*” [DR of “Description”]
- To express an opinion: “*in my opinion auditory hallucinations lead to school difficulties*” [DR of “Opinion”]

The above examples show how dialogic analysis is not related to what is said, and which (semantic) value it has for the interactants, but rather on how it is said, and which impact that specific modality has on the interactive process [24].

Each DR, in fact, has a specific numerical value, expressed in terms of Dialogic Weight (henceforth “dW”). dW, in turn, is related to the particular rhetorical-argumentative properties of each DR, and it indicates its potential to contribute to the discursive interactions [26].

The higher the dW, the more the DR promotes a generative discursive interaction, i.e. an interaction characterized for being possibilistic and based on the use of recognisable and shared elements. Conversely, a low dW accounts for language use modalities characterized for generating discursive interactions that pose themselves as a matter of fact, certain, and that are built through the use of personal and absolute references. Thus, dW provides researcher and practitioner with a measure of the impact of the language use modalities adopted to make sense of AVHs, allowing for comparable results among different narratives, both in cross-sectional or longitudinal studies.

By virtue of their dW, Dialogic Science organize the different DRs in a semi-periodic table (see [27]), distinguishing them in three typologies:

- *Stabilization DRs:* this group of DRs tend to rely on absolute references and personal perspectives, reducing the possible interactions with alternative narratives. For this reason these DRs have low values of dW. On an interactive level, these DRs promote the maintenance of a certain state of things, which is configured as a matter of fact.
- *Generative DRs:* these DRs are based on shared elements and possibilistic language use, which fosters interaction and collaboration between interlocutors. For this reason they are characterized by high levels of dW. These language modalities generate flexible discursive configurations that encourage the generation of new narratives and allow for changes in the construction of sense.
- *Hybrid DRs:* these DRs can assume either a Stabilization or Generative valence, depending on the other DR they’re interacting with.

Relying on these characteristics, we drew a connection between the linguistic interactions promoted by a certain DR and the construct of stigma. We drew from the symbolic interactionism tradition [28, 29], which aligns with Goffman [30]’s interpersonal definition of stigma^2^ to examine the interpersonal dynamics engendering stigma.

Hence, following the theoretical references of Dialogic Science, stigma consist in an interactive process that gradually reduces the possible “narrative twists” in the biography of a person [23]. Vice versa, under this conception, discursive productions oriented in terms of health are the ones promoting an active participation for each actor involved: triggering an interaction where all the roles have the opportunity to contribute with their resources to generate a biographic story opened to possibility [32].

In this sense, the more the DRs used in the interactions about AVHs are characterized for a low dW (Stabilization DRs), the more that discursive configuration will promote stigma and discrimination towards this theme. This is exactly because the narrative process will be characterised for creating a discursive configuration about AHV posed as a matter of fact, immutable and using personal references: hindering other interactants to contribute to the configuration and, thus, allowing them to change it. The following sentence exemplifies this typology of discursive modalities: *“Voices are a constant insult. They speak to each other and repeat”* [DR of “Certify Reality”]. Vice versa, a high dW indicates the use of language use modalities promoting the possibility for other “contributions” and, thus, the change of the configuration. Comparing the previous sentence with *“At first they insulted me, and it could happen that they started talking to each other, now they are phrases or words that are repeated in a loop”* [DR of “Description”], it’s possible to observe how a similar content (the insults) can be conveyed in more generative ways: reducing the absolutisation of the narration and posing it in more possibilistic terms. This way the content “insults” is conveyed as “one among the other elements” constituting the biographical story of the hearer, without going to exhaust the same.

**Fig. 1:**
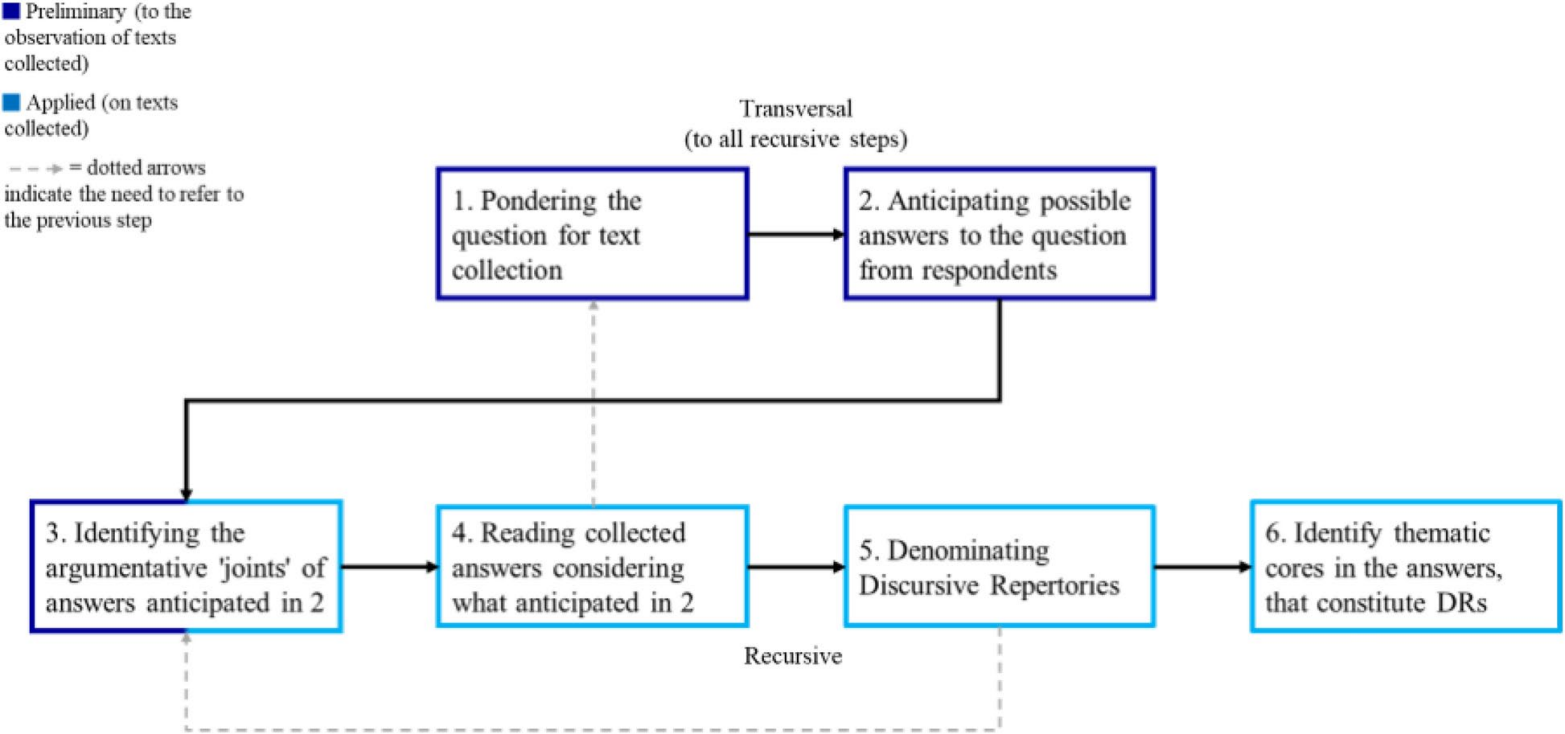
MADIT’s procedure for text data analysis

To perform the text analysis of DRs, we employed MADIT’s procedure, which consists of six sequential steps, as depicted in Fig. 1. The first two steps labeled as ‘transversal’-performed only once-and the subsequent four as ‘recursive’-requiring application to each individual response text. While the steps are linear, steps 3, 4, and 5 necessitate referencing earlier stages. Steps 3 and 4 revisit step 2 to aid researchers in adopting the respondents’ perspectives. This approach facilitates anticipation of potential responses and discursive strategies, enhancing accuracy and efficiency in steps 5 and 6. Additionally, step 5 revisits step 3 to maintain an awareness of common language uses pertinent to the topic. The identification of argumentative ‘joints’ and the assignment of corresponding DRs occur concurrently within MADIT, enabling analysts to immediately recognize how language is used and determine the appropriate DR. Finally, to keep track of responses’ thematic cores (step 6), we devised a set of “archipelagos of meaning”, i.e. thematic micro-categories generated according to the research objective (for the complete list of the “archipelagos of meaning” used for the research see Appendix-C).

Following the application of MADIT’s step to all research texts, these are processed using the software D.I.Ana., which organises the data and automatically calculates the Dialogic Weight [24]. We remark how Dialogic Science, in addition to the analysis of the content, allows us to assess and anticipate the implication that a certain rhetorical modality can have on the discursive interaction, leveraging the capability to measure narratives through DRs dW (we exemplify more this in Results and Discussion sections).

### Description of the questionnaire

To gather the community AVHs narratives we devised an ah-hoc built questionnaire covering 4 main areas of investigation, specifically:

- Area 1: Describe the discursive configuration regarding the voices and episodes in which the voices were heard.
- Area 2: Describe the discursive configuration related to the implications and challenges of hearing voices.
- Area 3: Describe the discursive configuration regarding the management of the implications and challenges of hearing voices.
- Area 4: Describe the configuration of roles/services used in managing the implications and challenges of hearing voices.

The questionnaire has been administered to 4 different groups of respondents, distinguished by their role with respect to the topic of AVHs, namely:

- Hearers: all those who for a certain portion of their life, more or less extended, have heard voices in the first person.
- Relatives and Friends: the roles that, during their life, have had the opportunity to interact with the hearers of voices, as relatives or friends.
- Professionals: the roles that find themselves interacting with respect to AVHs, as professionals in the management of the topic (such as psychiatrists, psychologists, nurses, social health operators, etc.)
- Externals: all the other roles in the community that responded to the questionnaire without falling within one of the previous categories.

The protocols varied based on role, altering both quantity and type of questions, resulting in four distinct versions (see Appendix-B). Primarily consisting of open questions, each closed question was paired with a subsequent open question to make explicit and deepen the content retrieved with the close one.

**Fig. 2:**
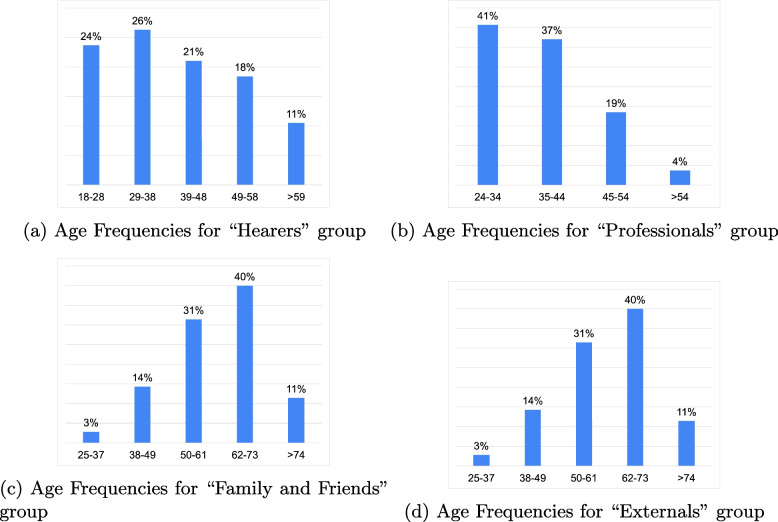
Absolute age frequencies distribution per group

The survey was conducted online, where participants, post-consent, responded to questions determining their role and corresponding questionnaire. “Associazione Nazionale Sentire le Voci”, assisted in spreading the survey. Thanks to the network of capillary relationships maintained by this association, the research has managed to involve a total of 268 respondents. Table 1 and Fig. 2a, b, c, d depicts the demographic characteristics of the sample, divided by the different roles.

**Table 1:** Descriptive statistics of the sample of respondents

	Hearers	Professionals	Relatives and friends	Externals	Total
**Participants**	53	46	44	125	268
**Sex**					
M	16	8	8	18	50
F	35	33	32	90	190
N/A	2	5	4	17	28
**Age Groups**	Fig. 2a	Fig. 2b	Fig. 2c	Fig. 2d	
N/A	17	4	6	8	35

## Results

### First research area: voice description

This section explores the community’s language use in discussing “hearing the voice” and actual instances of voice hearing. Information on onset age, voice count, and characteristics was collected using content-based questions. Open-ended questions further probed these areas and gathered narratives, like first voice-hearing experiences.

**Fig. 3:**
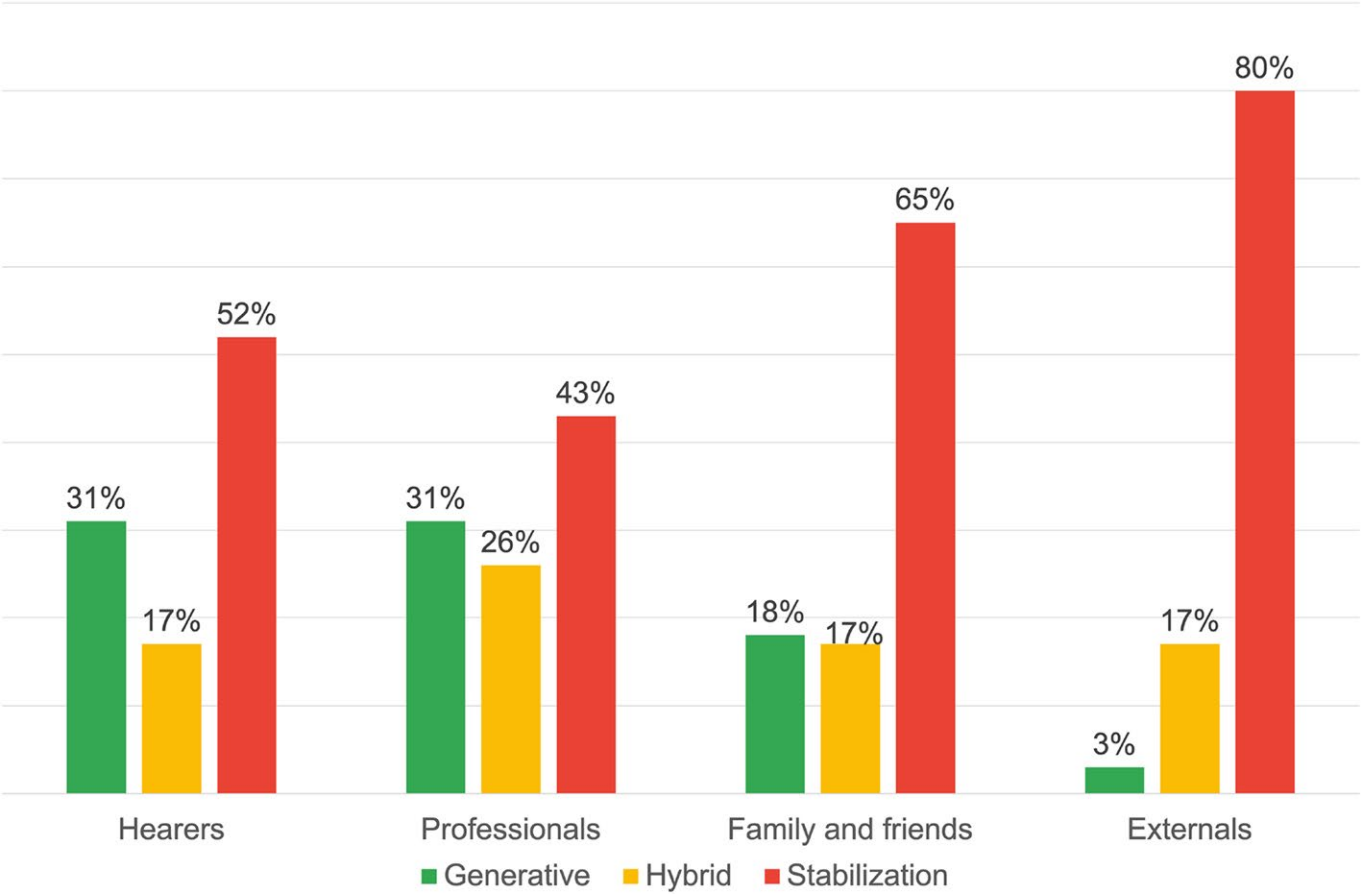
Distribution of DRs typologies for the first research area: voice description

DRs of Stabilization are predominantly used by the “External” respondent groups (80%) and “Family and Friends” (65%), at a notably higher frequency compared to the other groups (see Fig. 3). These narratives respond to questions like “how would you describe the voices this person hears?” and generate answers such as “sounds, noises”, “traumas that speak”, or “signs of fate”. These language use modalities are based on personal criteria and absolute responses, and portray AVHs as an unalterable fact. Interactions based on this “narrative style” can potentially lead to stereotyping and, if the contents are semantically negative, stigmatization of AVHs.

In contrast, “Voice Hearers” and “Professionals” groups extensively use Generative DRs (both at 31%). These texts are generated employing recognisable elements, allowing for an AVHs convergence of understanding. Examples include:

- *“One user said that he was in front of the TV and that the people he saw were talking to him, then coming out of the TV and commenting on what he was doing.”*
- *“I was going to work in the car and I was afraid that I wouldn’t be able to provide for my new family and the Male Voice told me that I could never make it on my own.”*

These two examples show how negative connotations of AVHs can be conveyed in a generative way, i.e. generating a highly shareable scenario. These texts string together various elements in a strictly descriptive logic, free from personal theories or interpretations. The value attributed to the narrative elements is explicit, so that the interlocutor can interact and create a common reality. This way, even with “negative” contents, this DR promotes configurations where all community roles can interact based on the same references, contributing with their perspectives. Thanks to these interactions the motion of the discursive process is increased, countering typification and stigmatization.

#### Voice onset

**Fig. 4:**
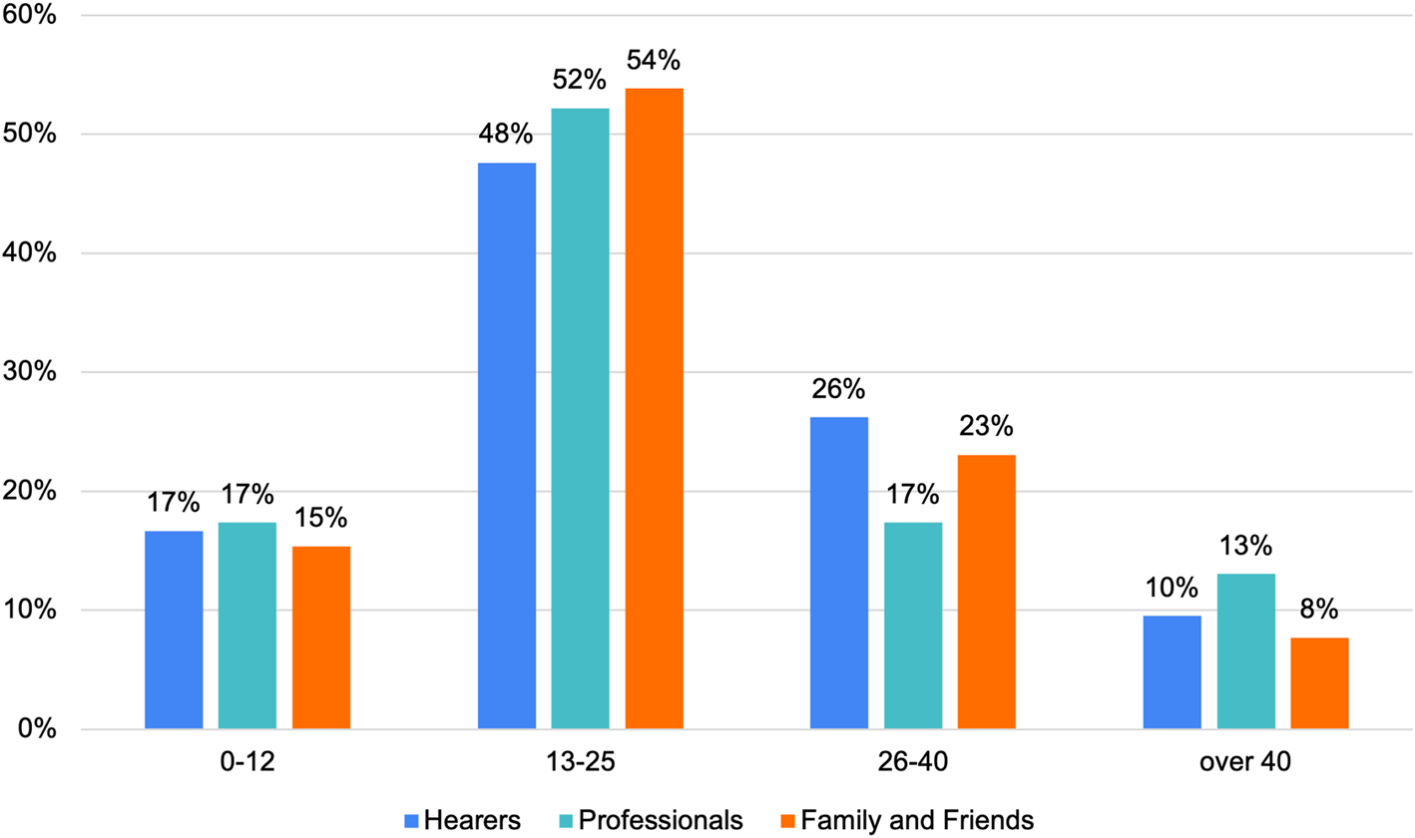
Age onset

In this section, we examine Voice Onset. Most participants can identify the Voice’s first emergence, namely: 80% of Hearers, 78% of Professionals and 95% of Family and Friends. On this regard, Fig. 4 show a common onset trend within the first 25 years.

**Fig. 5:**
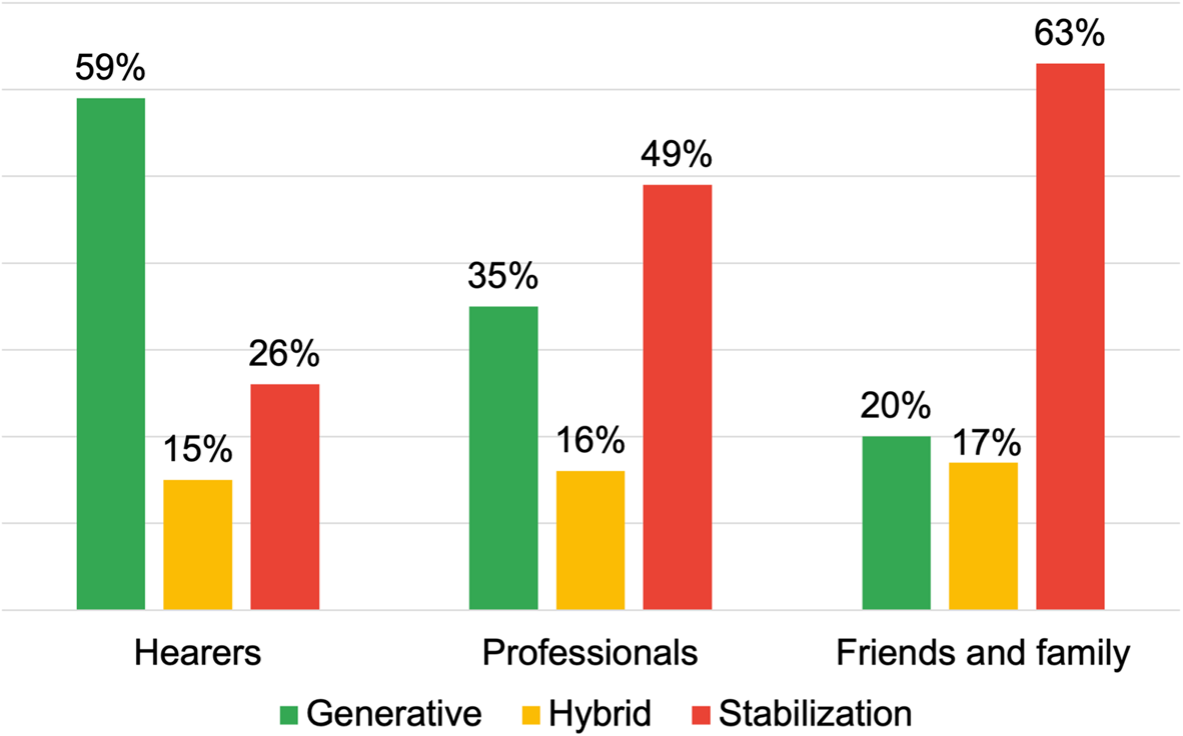
Distribution of DRs typologies for the research area: voice onset

Figure 5 provides information on the process-oriented inquiries, finding a high prevalence of Generative DRs among the “Hearers” (59%), generating texts like:

- *“I heard an outside voice as I read Dylan dog saying “you must die”, I went to the cafeteria I took a knife and began to slit my wrists. Then I recovered, as if waking up from a dark world, I went to ask my colleague for help. He called the ambulance and I passed the night in psychiatry”.*

Also in this case the discursive modality is characterized by the absence of personal assumptions or values, enabling the interlocutor to engage with the presented scenario and contribute to its development. Additionally, the discourse’s content is transmitted using a logical structure that situates the appearance of voices as one among multiple possible events in the listener’s life story. The language modality employed here is not intended to concretely delineate the manner in which voices emerge; instead, it facilitates an open-ended narrative progression, allowing for a multitude of potential developments, thus contrasting typification and stigma.

Table 2 depicts the results of the processual analysis of the text generated with respect to this dimension. Across different roles, particularly in the “Hearer” group, the emergence of AVHs is experienced as a solitary biographical moment in a private setting. “Professionals” and “Family and Friends” often report negative associations with AVHs’s origin, highlighting the stressful interactions with others and the negative effects of the voice. The answers of these groups allow to observe how AVHs’ insurgence can be conveyed through Stabilization DRs. *“I believe it was certainly terrible for her”*, for instance, negatively frames AVHs emergence as an absolute reality. These absolutist rhetorics tend to reduce the development of alternative discourses, leading to the stereotypical configuration of AVHs and people experiencing them.

**Table 2:** Processual analysis results: voice onset

	Hearers	Professionals	Family and friends
**Discursive Repertories**	Description: 58.82%	Description: 35.06%	Certify Reality: 28.13%
	Certify Reality: 14.12%	Certify Reality: 29.87%	Description: 20.31%
	Specification: 12.94%	Specification: 9.09%	Judgement: 10.16%
**Dialogic Weight**	7.1 dW	5.0 dW	3.3 dW
**Arcipelagos of Meaning**	Private Setting	Private Setting	School
	Solitary experience	Relatives	Solitary experience
	Defined Identity of the Voice	Stressful interaction with others	Negative Voice Implications

#### Voice in everyday life

**Fig. 6:**
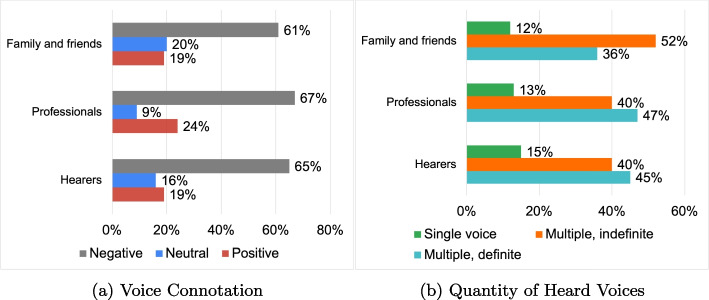
Closed questions results: voice in everyday life

**Fig. 7:**
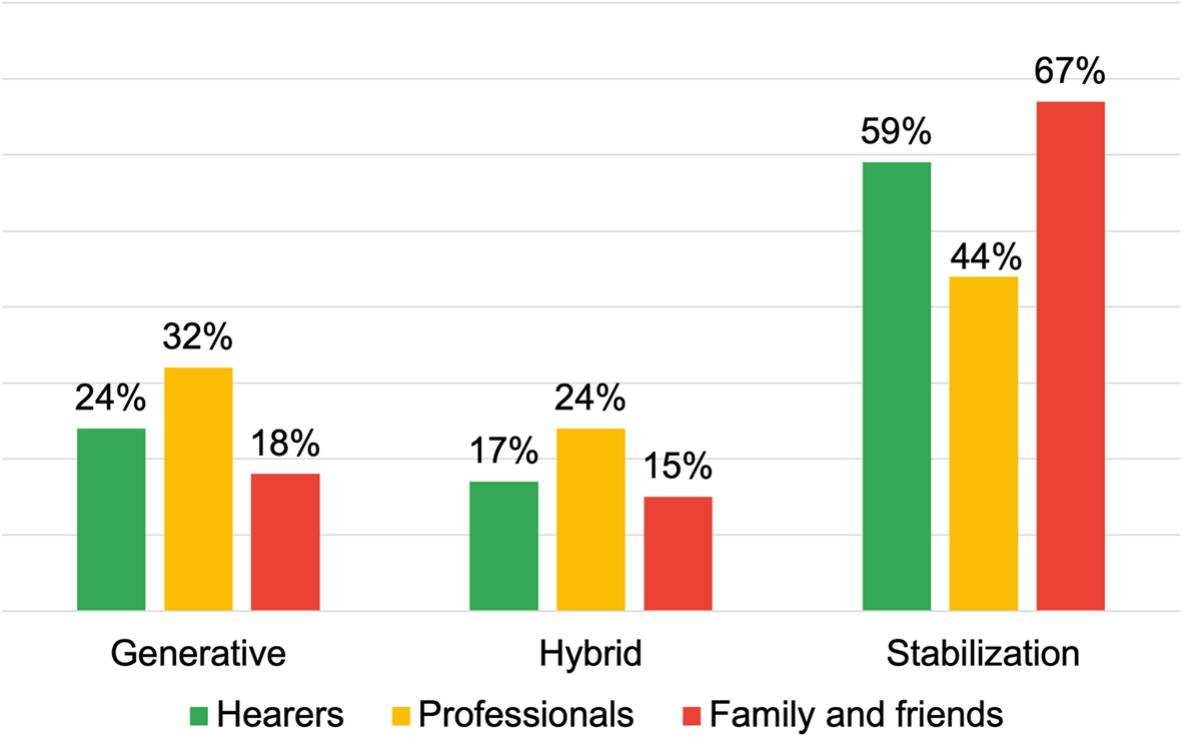
Distribution of DRs typologies for the research area: voice in everyday life

This subsection delves into current voice characteristics, assessing the number and overall sentiment of voices heard. Figure 6a indicates a prevalent negative characterization across research groups. Figure 6b shows a commonality of multiple, sometimes unquantifiable, voices. Figure 7 and Table 3, focusing on process questions, reveals “Hearers” and “Family and Friends” often employ Stabilization DRs with low Dialogic Weight (3.5dW and 3.2dW, respectively). In contrast, the “Professionals” group produced a more generative configuration (4,8dW), positioned mid-way on the continuum. In this regard, content questions revealed a general negative connotation of the voice; however, the process question showed different modes of conveying these contents. This highlights how the same elements can be conveyed through different discursive modalities. For example, the negative connotation of AVHs can be conveyed through DRs like “Judgment” with texts like:

- *“Negative, threatening, offensive, dialoguing, commenting, derogatory voices”.*

In the provided examples, the voice’s attributes are framed as an immutable fact. Not explaining the criteria for judging the voices as “negative, threatening, etc.” implies that the underpinning reasons remain implicit and not shared with the interlocutor, who then applies a personal understanding when using these elements. Interactively, thus, through the use of absolute rhetorics and personal criteria, the connotative elements promote stereotyped processes, in which both the hearer and interacting roles continue to perceive the voice’s negativity as a given fact.

**Table 3:** Processual analysis results: voice connotation

	Hearers	Professionals	Family and friends
**Discursive Repertories**	Certify Reality: 35.93%	Description: 31.95%	Certify Reality: 26.98%
	Description: 19.63%	Certify Reality: 25.56%	Judgement: 19.06%
	Judgement: 11.11%	Specification: 19.17%	Description: 17.63%
**Dialogic Weight**	3.5 dW	4.8 dW	3.2 dW
**Arcipelagos of Meaning**	Internal Voice	Negative Voice Implications	Negative Voice Implications
	Active Engagement of Other People	Negative and Judgemental Voices	Negative and Judgemental Voices
	External Voices	Prescriptive Voices	Prescriptive Voices

The “Professional” group provides useful examples of more generative ways to convey the criticalities of everyday life’s AVHs:

- *“Currently the patient reports the voice as a continuous whisper that occasionally turns into a scream. Initially, it was a clear voice telling her clearly to harm herself”.*

This narrative is constructed on neutral and shareable elements, allowing readers to form a clear image without personal references to fill in the meaning of certain terms. Moreover, the narrative contextualizes the description, presenting elements that could indicate a negative connotation of the voice as aspects of an evolving process. Conveyed in this non-absolutist manner, the contents open up the possibility of “saying something else” about the voice, pragmatically leading to the generation of new discourses on the subject, new directions in the individual’s biographical path, and potentially new management strategies for issues related to the voice.

### Second research area: voice implications

This research segment aims to uncover outputs about the effects (present or future) of voice hearing on interpersonal interactions. By combining multiple-choice and open-ended queries, it seeks to explore the integration of voice experiences into participants’ life narratives and the impact on their work, family, and social dynamics. Transversely to the groups, the implications of hearing voices are configured as factual realities.

- *“They totally influenced my school and my future.”*

The text exemplifies how language can exhaust the space for other possible narratives regarding AVHs role in people’s biographical path.

This point is echoed in responses from groups like “Family and Friends”, who, when asked “What do you think are the aspects of a person’s life that are most influenced by hearing voices?”, replied with:

- *“In my opinion, all aspects of life.”*

When interactions are based on discursive production like this, typification processes are promoted: voice’s pervasiveness in participants’ lives is seen as a given, hindering the creation of alternative narratives about its value in the daily lives of both the hearer and others involved. Moreover, the absoluteness characterizing these texts frames the criticalities as an element that will continuously be present in the hearer’s life, even in future perspectives.

**Fig. 8:**
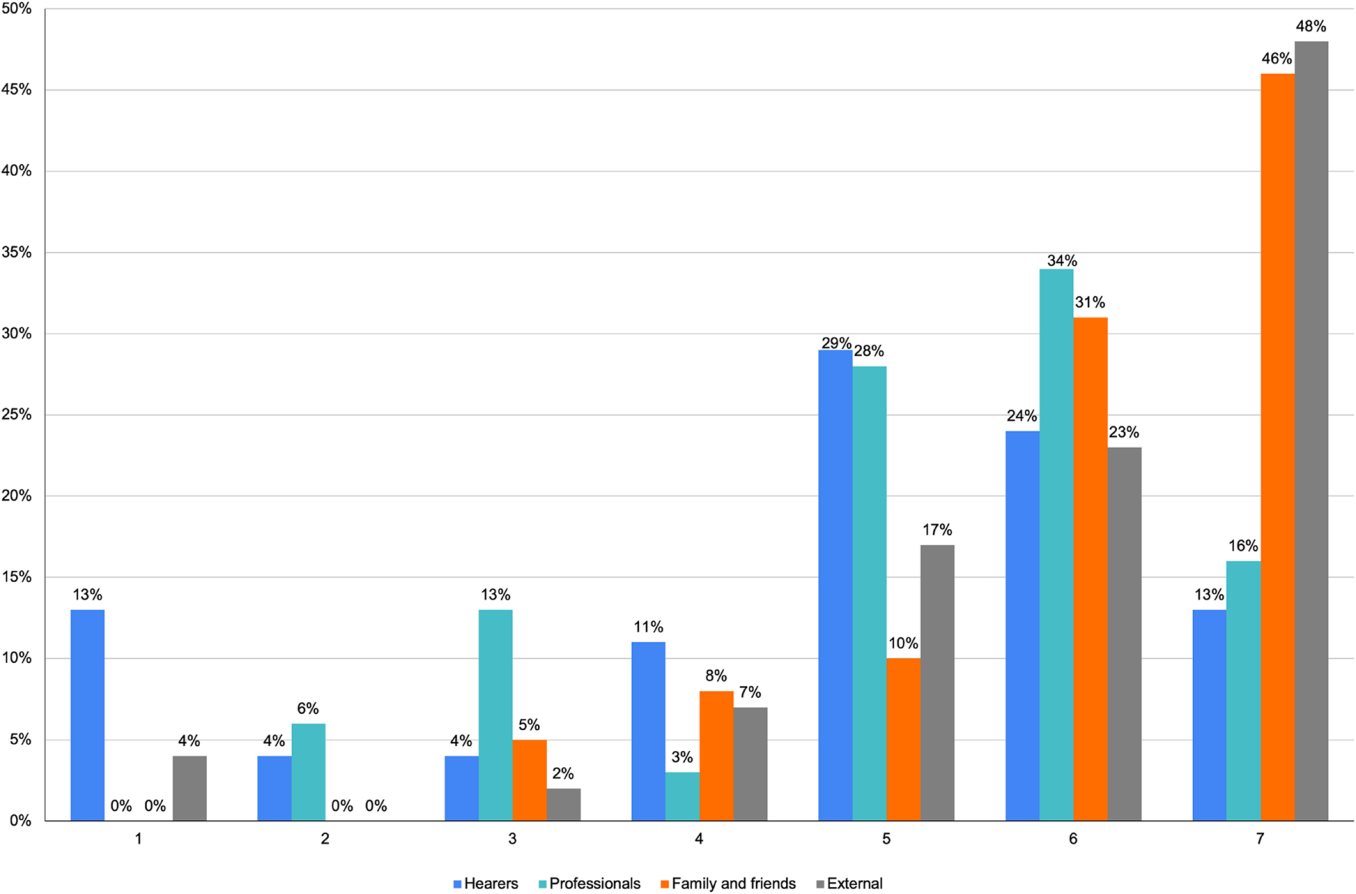
Likert voice impact ratings (1=min; 7=max)

Figure 8 presents a Likert scale evaluation (1-7) of the voice’s impact on hearers’ lives. Across all groups, a high impact is reported, with most ratings falling between 5 and 7. The Likert evaluation was supplemented by open-ended questions for deeper insight into these scores.

Table 4, examining justifications for these scores, reveals a common theme: respondents generally do not pinpoint specific areas influenced by the voice. The theme “General context of the voice’s influence” appears frequently across all groups, with excerpts like:

- *“[the voice has an impact] when I am stressed, when I have to do something.”*
- *“I think [the voices] can affect any aspect and can vary from person to person” *

**Table 4:** Processual analysis results: voice implications

	Hearers	Professionals	Family and friends	Externals
**Discursive Rep.**	Certify Reality: 37.6%	Certify Reality: 35.8%	Certify Reality: 42.7%	Certify Reality: 42.1%
	Description: 19%	Description: 22.4%	Description: 11.1%	Generalization: 11.4%
	Judgement: 10.7%	Specification: 10.8%	Specification: 8.5%	Specification: 9.3%
**Dialogic Weight**	3 dW	3.4 dW	2.2 dW	1.1 dW
**Arcipelag. of Mean.**	General context of voice influence	Person facing difficulties	Relationships	Influential voice
	Person facing difficulties	Relationships	Person facing difficulties	Management Issues
	Limiting effect of the voice	General context of voice influence	General context of voice influence	General context of voice influence

These excerpts also reflect the respondents’ modes of framing the voice’s impact on daily life, mostly falling within the Stabilization DRs. In the examples, the voice’s impact is depicted as pervasive across all aspects of daily life without explicit criteria, keeping the discourse within a personal dimension.

Yet, the data indicate that the influence of the voice on daily life is not uniformly high. Among the “Hearers”, 32% rated the impact as medium or low (4 or below on the Likert scale). Open-ended questions revealed that this variation is marked by the content conveyed, but not necessarily by the modalities. Consider this excerpt:

- *“Because they help me a lot in living and relating”.*

**Fig. 9:**
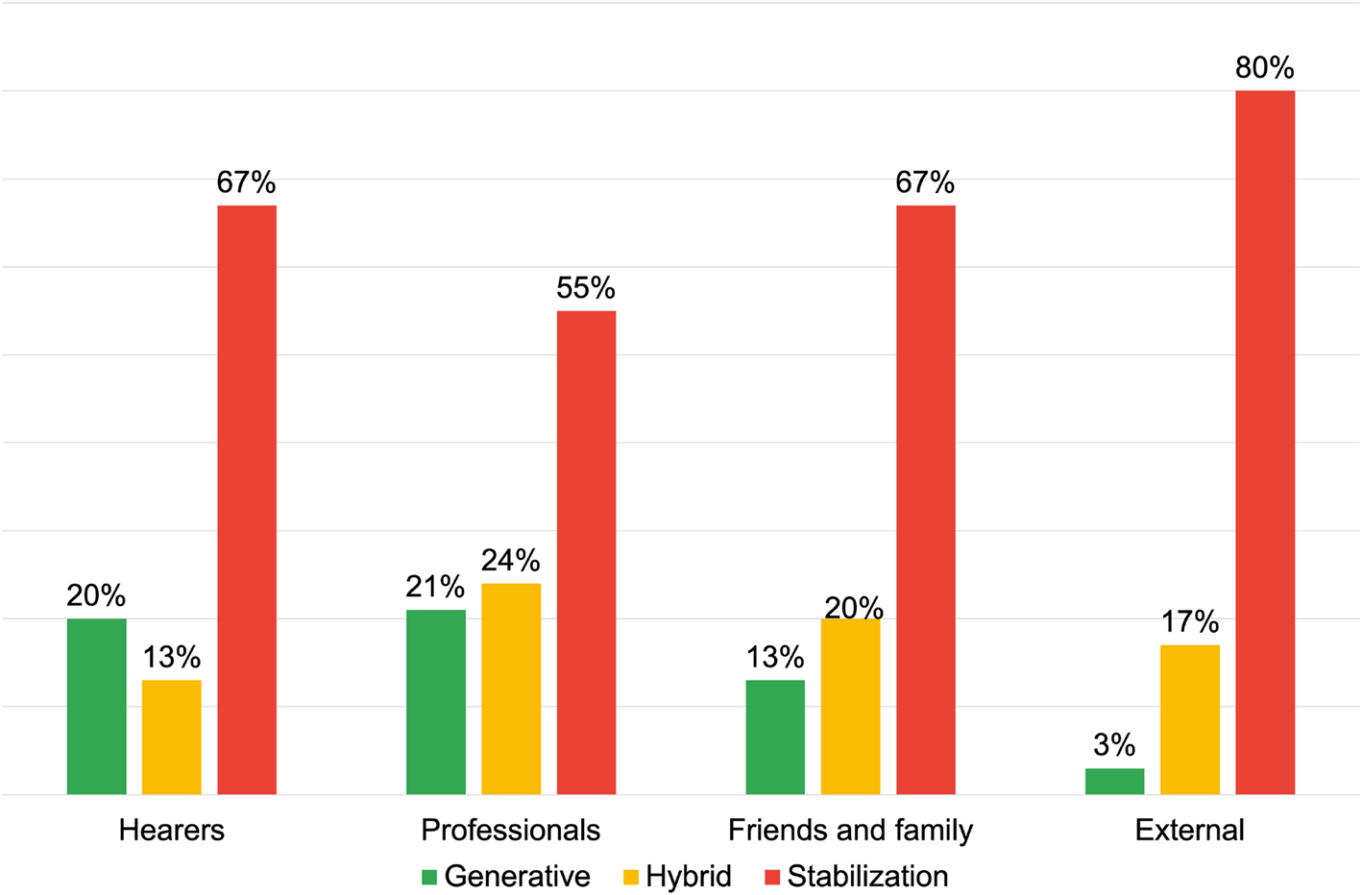
Distribution of DRs typologies for the research area: voice implications

Here, the voice is framed as a supportive element in daily life. At the same time, the discursive modality employed ties the content’s value to the respondent’s personal criteria, preventing the interlocutor from sharing the value of “they help me a lot in living” and using it towards a common scenario. Thus, even if semantically opposite to community narratives, these discourses limit their potential for changing and interaction the typification and stigmatization processes (Fig. 9).

### Third research area: voice management strategies

This section scrutinizes the management strategies for AVHs and their consequences. Content-based queries were utilized to assess the medical management aspect, focusing on psychopharmacological treatments and hospitalization. Subsequently, open-ended questions probed deeper into narrative constructions about medical management, while also inviting descriptions of non-medical management methods (see Table 5). Overall, the discourse predominantly exhibits a stabilization trend (2.35dW). On a semantic level, however, there are opposing positions.

- *“I can’t handle them.”*
- *“I manage easily by myself.”*

**Fig. 10:**
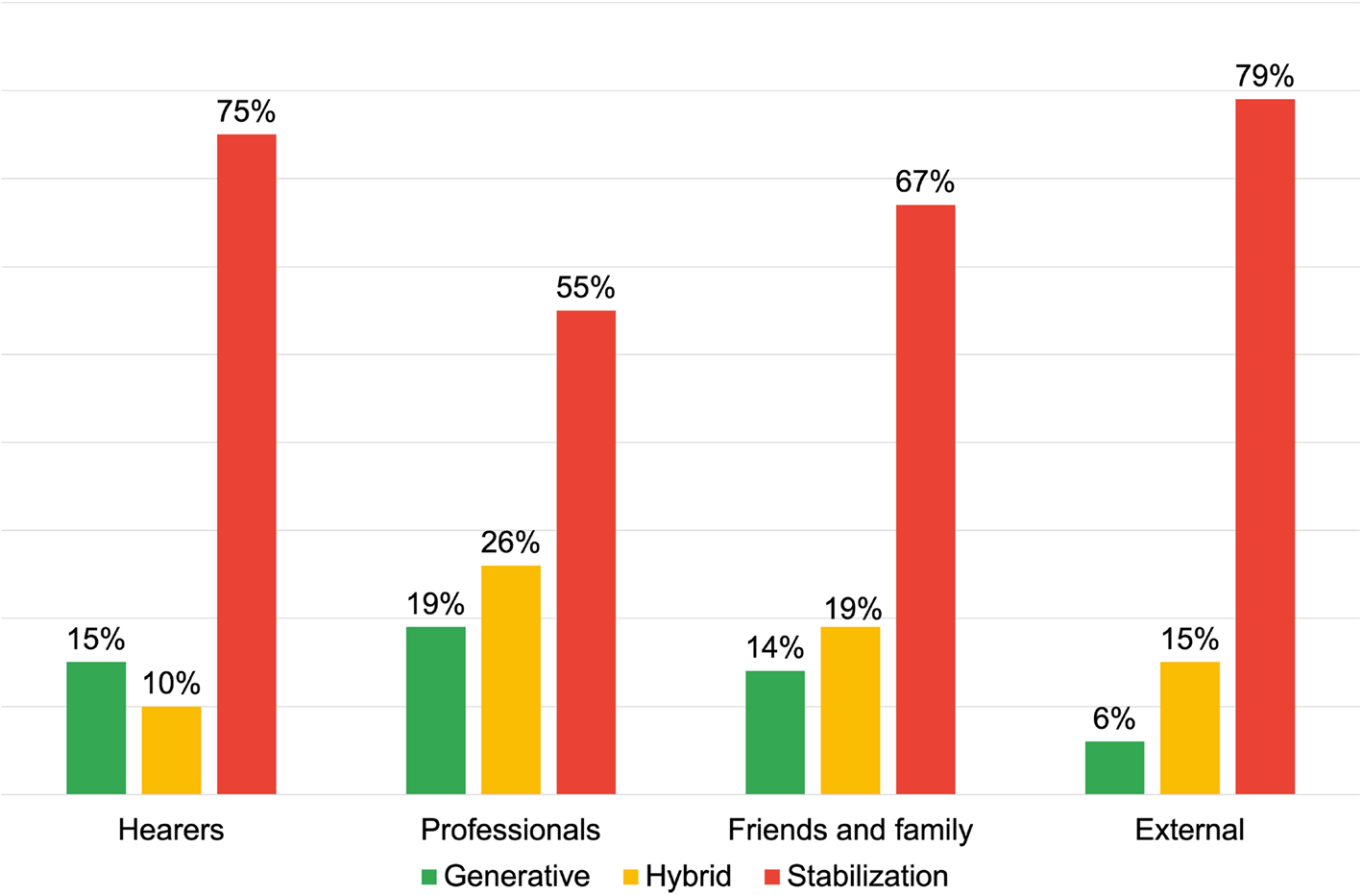
Distribution of DRs typologies for the research area: voice management strategies

Responses like the ones in the example represent two content-wise opposite examples, yet both frame the scenario as certain and unchangeable. The first scenario implies a definite inability to manage, while the second assumes successful self-management as a fact. These scenarios have potential critical implications (Fig. 10).

**Table 5:** Processual analysis results: voice management strategies

	Hearers	Professionals	Family and friends	Externals
**Discursive Rep.**	Certify Reality: 41.9%	Certify Reality: 31.5%	Certify Reality: 34.1%	Certify Reality: 47.3%
	Description: 14.5%	Description: 18.8%	Description: 12.4%	Generalization: 8.7%
	Justification: 8.9%	Specification: 13.4%	Specification: 9.7%	Specification: 7.9%
**Dialogic Weight**	3 dW	3.4 dW	2.2 dW	1.1 dW
**Arcipelag. of Mean.**	Self-Management	Self-Management	Self-Management	Listening and understanding from others
	Management inability	Listening and understanding from others	Management Issues	Management with Professionals
	Listening and understanding from others	Management with Psychologist	Management inability	Management with Psychologist

In the first (“Inability to Manage”), the manifestation of AVHs is always seen as problematic, with this perception extending into the present, past, and future. This absolutism limits the expression of alternative viewpoints, framing prompts delegation processes, where managing the difficulty is deferred to others, reducing the chance to develop useful management skills.

In the second scenario, since successful management is taken for granted, there’s a lack of anticipation for alternative strategies if usual methods fail. In unforeseen situations where personal resources are insufficient, this could lead to critical outcomes, affecting the biography, like hospitalization.

This last anticipation is of particular relevance since the most frequent theme across roles is “Self-Management”. Similar anticipations can also be applied to excerpts conveying contents related to the management of voices through the interaction with others, such as “Management with a Professional”, “Management with a Psychologist”, or “Listening/Understanding by others”.

Consider this example:

- *“The only way is to talk to someone who believes me and can give me advice to understand its meaning.”*

This text presents a scenario where reliance on others is the sole management strategy for the challenges of AVHs. This suggests potential difficulties, especially if such supportive roles are absent, leaving those involved vulnerable to uncertainty and risk. Moreover, even if supportive roles are consistently available, challenging situations may arise that are difficult to manage, leading to critical issues in the biographies of those involved.

Finally, various respondents resorted to Generative DRs, albeit less frequently. The text “*Some strategies they often use include writing their thoughts in a notebook, listening to music, or keeping the TV off*” delineates self-management techniques for AVHs, offering a detailed perspective and supplying elements conducive to interaction. This approach fosters a collaborative environment, empowering individuals to leverage the provided information as tools in managing voice experiences. These narratives, therefore, not only differ from the community roles’ delegation processes but also offer potentially valuable material for developing new management practices for AVHs.

#### Hospitalization and psychopharmacological treatment

This section delves into the impact of Auditory Verbal Hallucinations (AVHs) on hospital admissions and psychopharmacological treatment usage. As introduced, these aspects are often seen in the scientific discourse as indicative of AVHs being a psychopathological issue under medical purview.

**Fig. 11:**
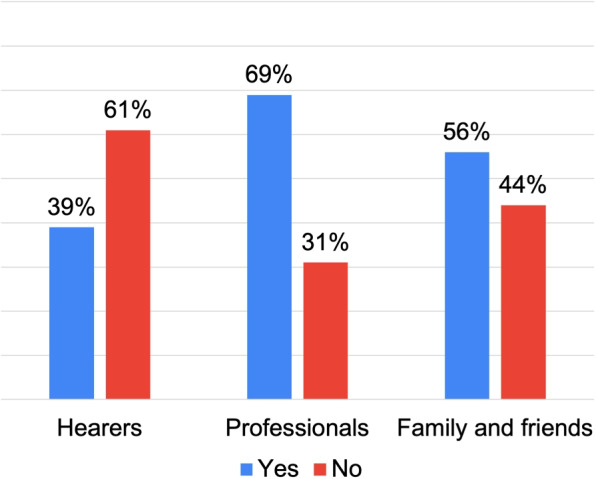
Closed questions results: hospitalization

Figure 11 shows that the “Professionals” and “Family and Friends” groups are more likely to report hospitalizations. In contrast, the “Hearers” group demonstrates a lower tendency for such interventions. These findings challenge the prevalent medical narrative, which typically links the emergence of hallucinations to hospitalization, as Mueser et al. [33] suggest.

**Fig. 12:**
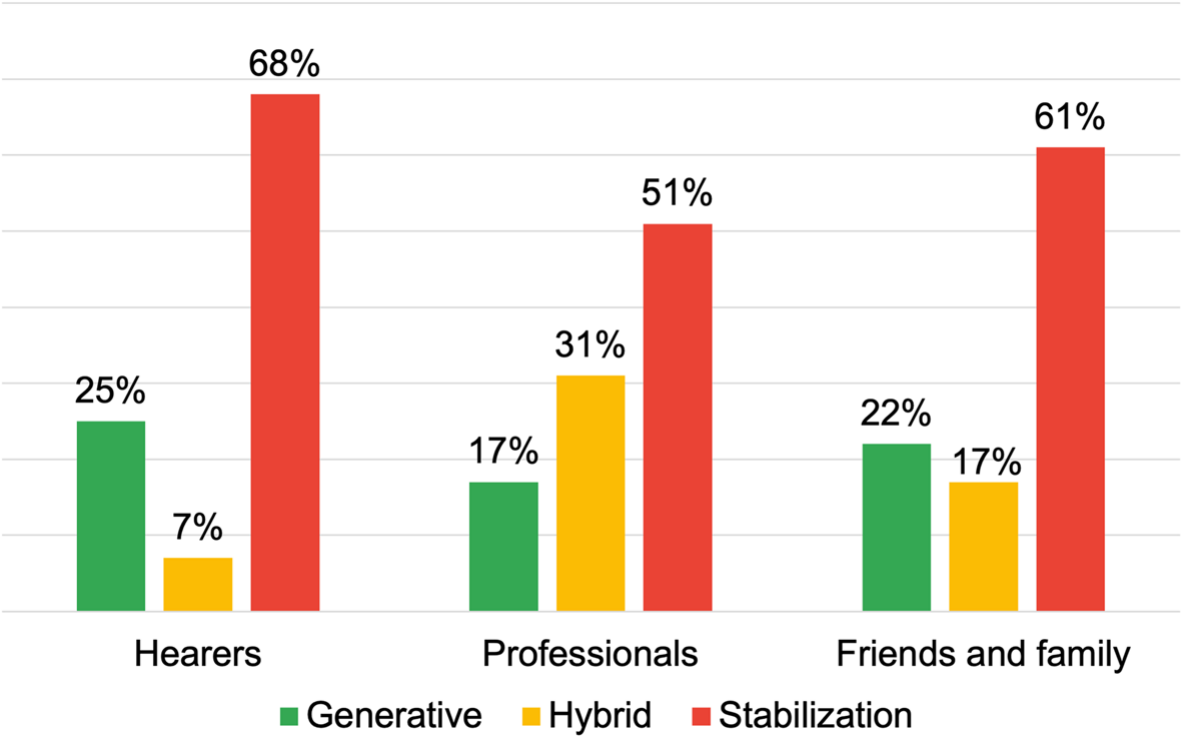
Distribution of DRs typologies for the research area: psychopharmacological treatment and hospitalization

Table 6 illustrates a consistent use of psychopathological terminology by all three roles in discussing hospitalization experiences. Notably, as shown in Fig. 12 “Professionals” mainly employ Stabilization and Hybrid DRs, resulting in a lower generative discourse (1.8 dW).

- *“[The user was hospitalized] during periods of severe discomfort [...]” (Stabilization DR) *
- *“[...] when the voices are no longer under control”* (Hybrid DR)

**Table 6:** Processual analysis results: psychopharmacological treatment and hospitalization

	Hearers	Professionals	Family and friends
**Discursive Repertories**	Description: 25%	Certifying Reality: 40%	Certify Reality: 31.7%
	Certify Reality: 21.4%	Specification: 22.8%	Description: 21.9%
	Judgement: 14.2%	Description: 17.1%	Specification: 12.2%
**Dialogic Weight**	4.7 dW	1.8 dW	3.8 dW
**Arcipelagos of Meaning**	Person facing a psychopathology	Person facing a psychopathology	Management Issues
	Management with psychiatrist	Stressful interaction with others	Person facing a psychopathology
	Management with Public Health Institutions	Suicide	Pharmacological treatment

The examples illustrate this: the first phrase sets a certain and absolute scenario, while the second supports it with specific details. The use of these rhetorics hinders the creation of relatable scenarios about hospitalization. For instance, the term value of “severe discomfort” is personal, which could lead to issues in interactions with others, such as hearers or their families, who might not agree with this characterization and oppose hospitalization decisions. Such disagreements could trigger critical consequences like involuntary hospitalization, leading to the development of typification processes.

In contrast, the “Hearers” and “Family and Friends” groups, with 4,7 dW and 3,8 dW respectively, frequently use Generative Discursive Repertories. An example is:

- *“I heard these voices saying my friends were in danger, I went to the emergency room claiming I was a medium and needed to be suppressed, and the psychiatrist admitted me here at the csm.”*

In this instance, the content also depicts a scenario leading to hospitalization. However, the used DRs allow for a deeper exploration of the situation, enhancing understanding about the subject, thus creating a different narrative configuration. This configuration of AVHs management fosters the development of inclusive strategies that, by using recognisable and relatable references, recognize and value contributions from a range of roles, promoting their collaborations and countering the emergence of stereotypes or stigma.

**Fig. 13:**
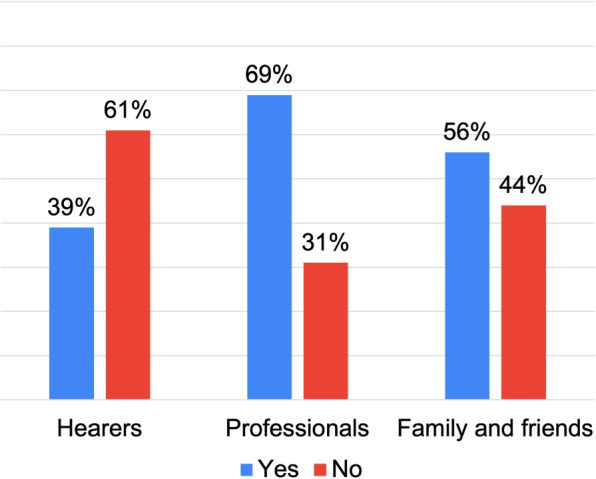
Closed questions results: psychopharmacological treatment

Finally, Fig. 13 reveals varying patterns in psychopharmacological use among different roles. While “Professionals” and “Family and Friends” show a clear inclination towards medication use (especially the former), the “Hearer” group is evenly split between users and non-users. A key takeaway from this data, thus, is that psychopharmacological intervention is not an inevitable consequence of AVHs.

### Fourth research area: interactions with community roles

**Fig. 14:**
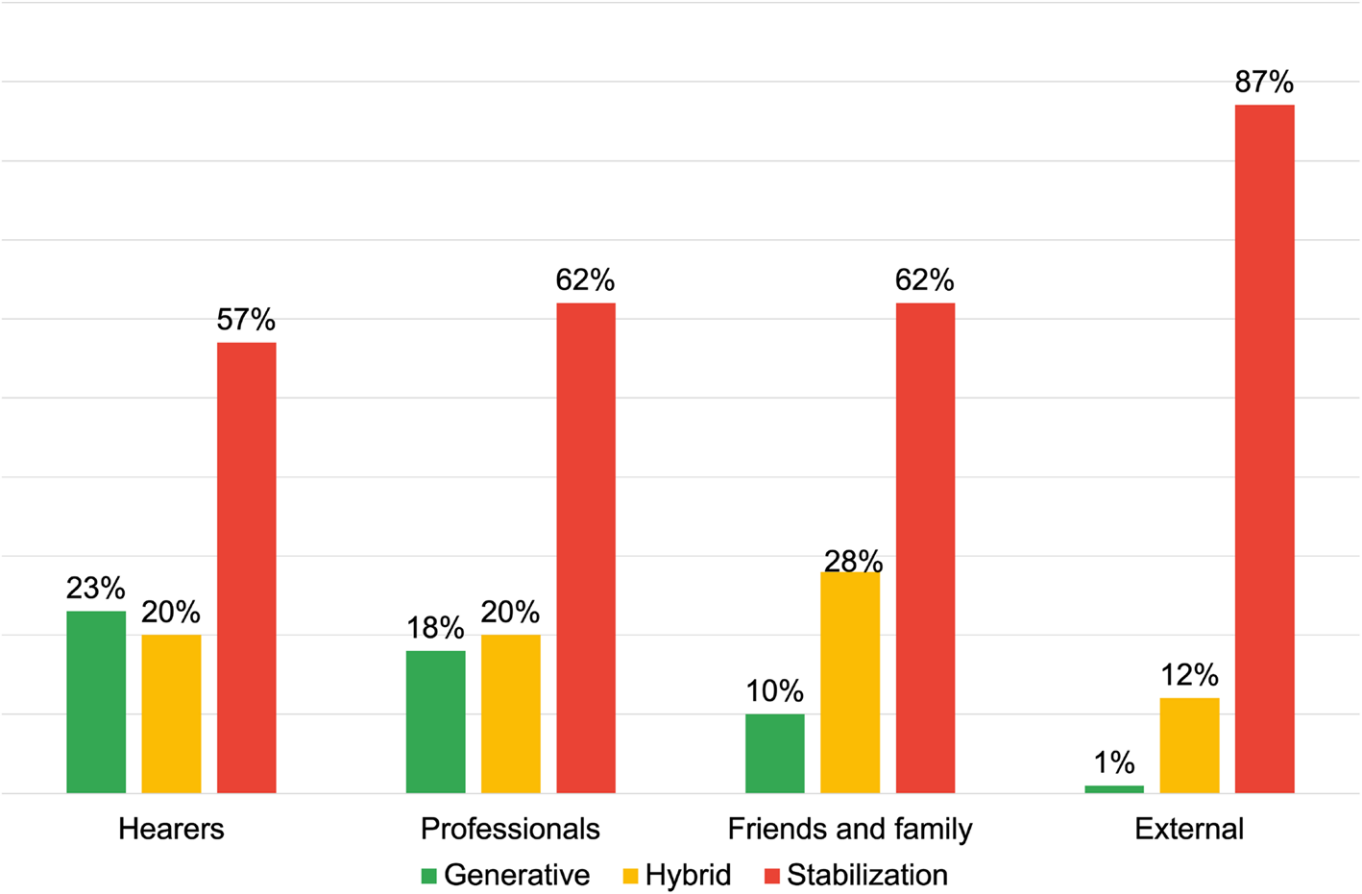
Distribution of DRs typologies for the research area: interactions with community roles

The fourth research area investigates respondent language in defining institutional services’ role in AVHs. Figure 14 indicates “Professionals” and “Family and Friends” predominantly adopt a stabilization discourse approach (see also Table 7).

**Table 7:** Processual analysis results: interactions with community roles

	Hearers	Professionals	Family and friends	Externals
**Discursive Rep.**	Certify Reality: 30.9%	Certify Reality: 37.1%	Certify Reality: 25.7%	Certify Reality: 58.5%
	Description: 22.4%	Description: 16.5%	Evaluation: 14.9%	No Answer: 17.2%
	Specification: 7.3%	Specification: 11.3%	Judgement: 10.8%	Specification: 3.9%
**Dialogic Weight**	3.7 dW	2.6 dW	2.5 dW	0.7 dW
**Arcipelag. of Mean.**	Management issues	Management through Healthcare Institution	Management with Association	Management through Healthcare Institution
	Management with Private	Management with Private	Management with Private	Management with Private
	Management with Association	Management issues	Management issues	Other

However, distinct trends are observed for “Hearers” and “External” groups, with the latter heavily relying on Stabilization DRs (87%), leading to a notably low Dialogic Weight of 0.7 dW.

- *“Psychiatric service.”*
- *“Psychologist.”*
- *“Doctors”*

The provided examples illustrate how language is used to define in a general and vague way the roles responsible for managing these implications, based on implicit and personal criteria. This promotes delegation of responsibility to institutional services. At the same time, the modalities through which this management could take shape are not made explicit, creating a fragmentation of medical praxis.

Conversely, the “Hearers” group, despite a general tendency towards stabilization (3,7 dW), exhibited more Generative DRs. Consider this example:

- *“I asked my sister for help, and she directed me to a psychologist who then advised seeing a psychiatrist.”*

This language use modality, in response to*“Why did you turn to the roles you indicated? Explain”*, is characteristically descriptive, not relying on personal conceptions or judgments. This way interlocutors are predisposed to engage with the offered content, thereby encouraging the generation of reflections and anticipations based on the presented information. This generative production, in fact, employs discursive processes that assign to the other not a predefined definition, but the role of a legitimate interlocutor for continuing to create a mutually beneficial reality.

Comparing this approach with the responses of the “External” group, it emerges that, while both end in the conclusion of contacting healthcare roles, the manner in which this content is delivered leads to the creation of completely different scenarios and interactive processes.

#### Using roles to manage critical voice implications

**Fig. 15:**
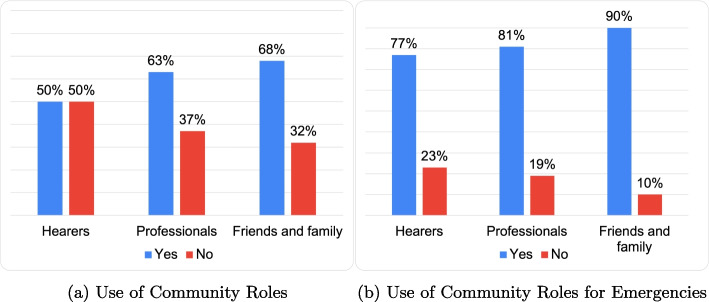
Content analysis results: using roles to manage critical voice implications

This section examines the interactions with institutional roles in managing AVHs. Analysis of Fig. 15a and b reveals a substantial variation in interactions depending on the request type, especially in crisis scenarios.

**Fig. 16:**
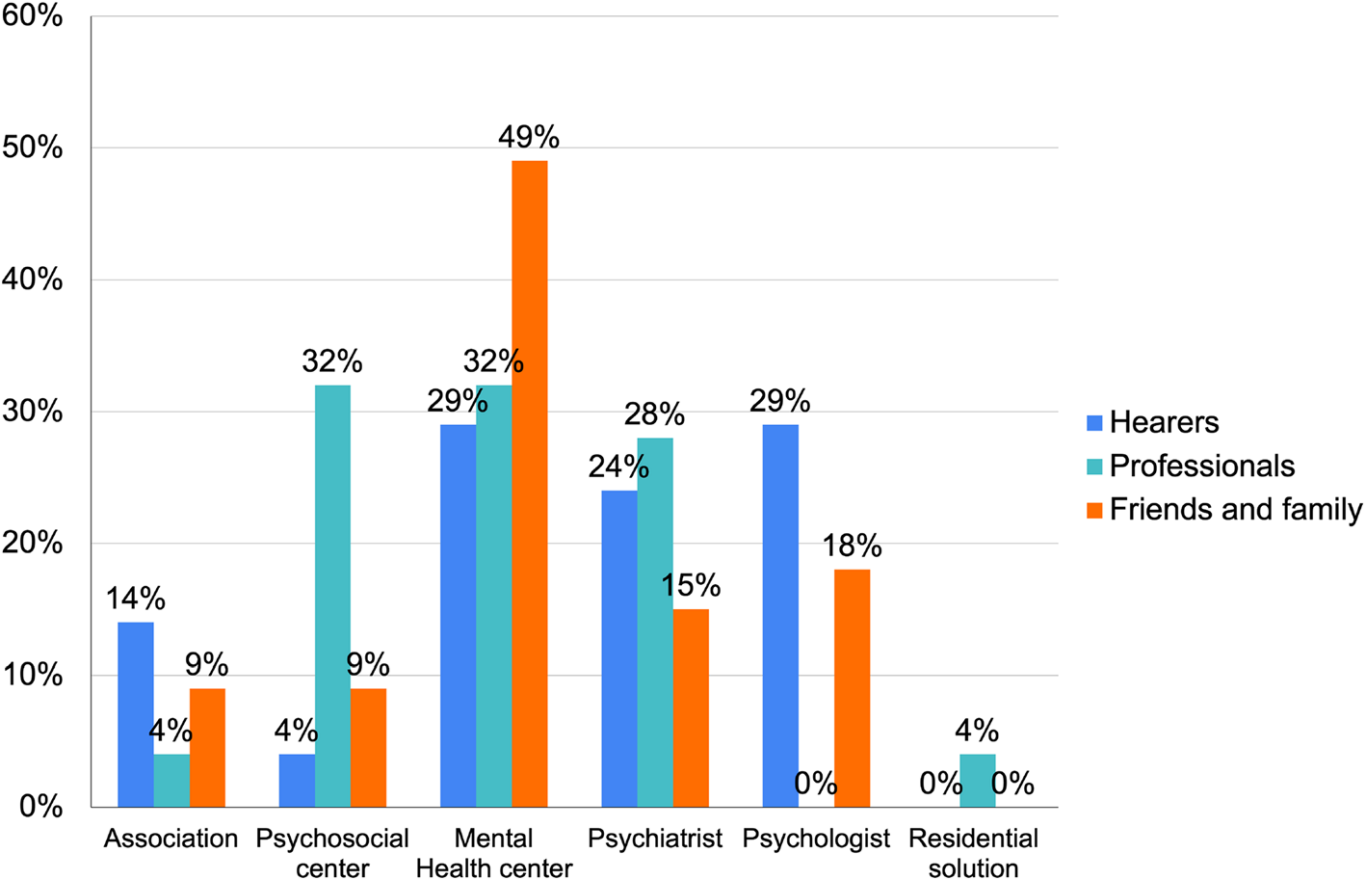
Categories of contacted roles

Predominantly, respondents in critical situations reached out to local services. Figure 16 further shows these services are mainly healthcare-oriented. Thus, it’s evident that healthcare professionals are often the first point of contact in emergencies, suggesting that delayed intervention could impact the individual’s health outcome.

**Fig. 17:**
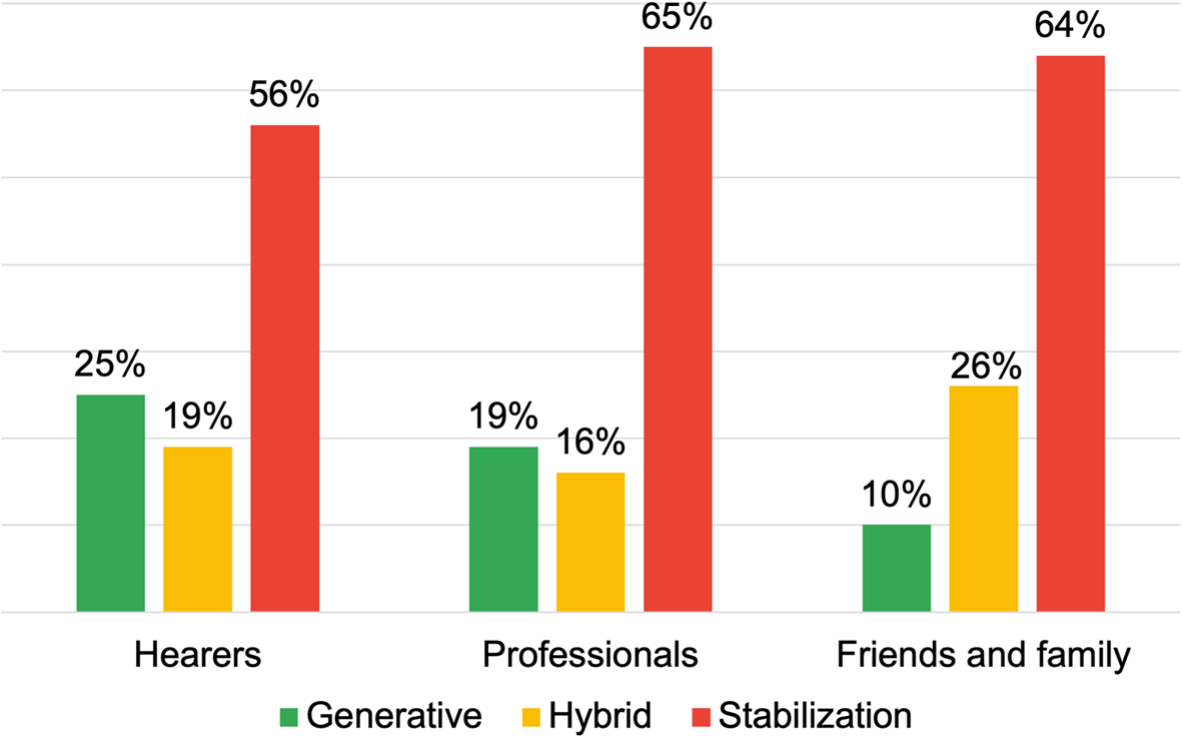
Using roles to manage critical voice implications

Table 8 and Fig. 17 reveal that the configurations are predominantly created through Stabilization DRs. Specifically, the most utilized DR is “Certify Reality,” which, in contrast to the explicit theories behind choosing a specific community role, produces statements like:

- *“I needed external help.”*

This extract shows that narratives is closely tied to personal theories and references, which hinder the audience’s engagement with the presented content. This aspect should be considered alongside data indicating that one of the most frequently used topic area relates to “Crisis in Management,” with statements like:

- *“Zero help, zero understanding.”*
- *“the psychiatrist didn’t help me at all.”*

Hence, on one hand, the relationship with community roles is defined in terms of crisis and as a lack of efficacy; on the other hand, the narrative about unmet needs or requests unfold in personal ways. These last, being strongly related to personal references, impede the initiation of processes that could change the management of these crises, further reducing the effectiveness of these services.

**Table 8:** Processual analysis results: using roles to manage critical voice implications

	Hearers	Professionals	Family and friends
**Discursive Repertories**	Certify Reality: 31.06%	Certify Reality: 37.66%	Certify Reality: 26.57%
	Description: 25%	Description: 18.18%	Evaluation: 14.69%
	Specification: 9.09%	Specification: 10.39%	Judgement: 11.19%
**Dialogic Weight**	3.9 dW	2.7 dW	2.5 dW
**Arcipelagos of Meaning**	Management issues	Management with Public Institutions	Management with Association
	Management with Private Psychologist	Management with Private	Management with Private Psychiatrist
	Management with Private Psychiatrist	Management issues	Management with Private Psychologist

The group of “Hearers” is highlighted for often using Generative DR (25%). Consider the statement:

- *“I discovered the SLV association a while ago, I was drawn to the people who found a way to coexist that helps others and I approached them.”*

Here, the engagement with the Association and the reasons for embarking on this path are presented in a more relatable logic. The narrative is characterized by providing the audience with relatable elements that enable them to engage with the presented scenario.

Free from personal judgments or values, this narrative allows participants to use it as a “common convergence element,” initiating new interactions with contributions from all parties involved. Due to the neutrality of the described criterion, the generated text has the potential to be used, for instance, to share objectives with the audience, thus creating a common horizon to which both the community service (role) and the audience can turn together to structure support (such as the development of the competencies that sparked curiosity).

#### Not using roles to manage critical voice implications

**Fig. 18:**
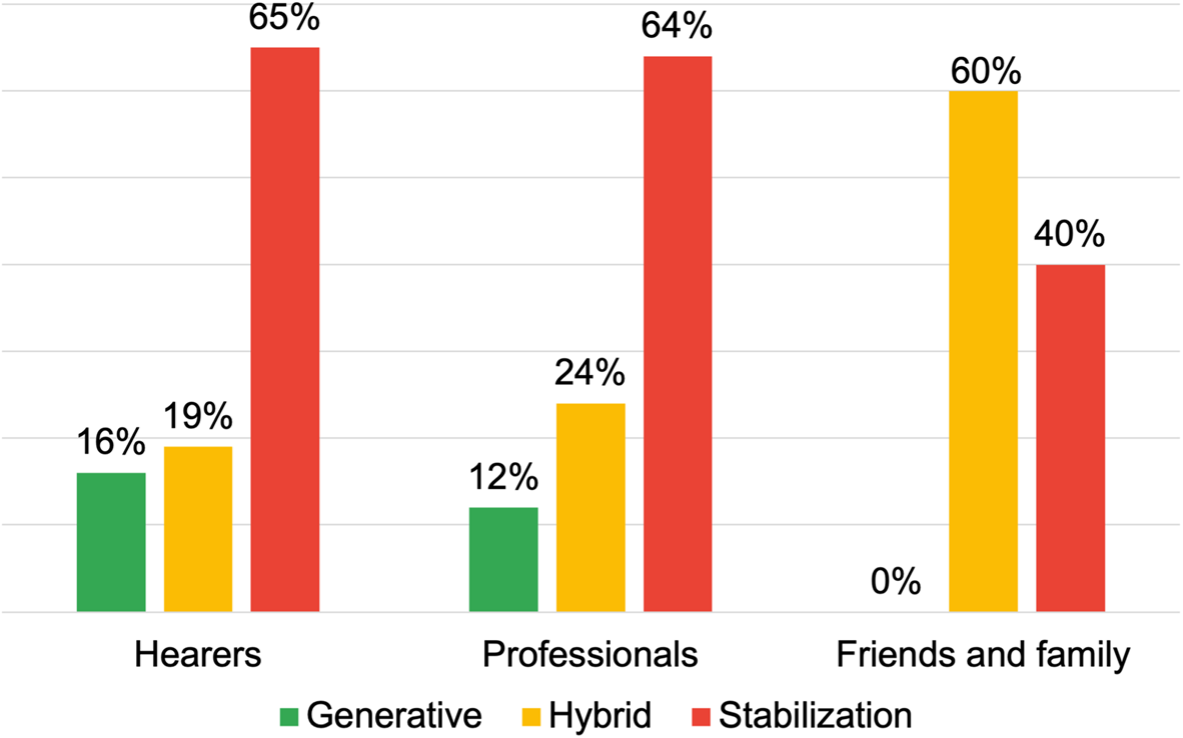
Not using roles to manage critical voice implications

Table 9 and Fig. 18 reveal how both “Professionals” and “Family & Friends” predominantly use Stabilization DRs, resulting in low dW discursive configurations (2.7 dW and 2.2 dW respectively). In this sense the reasons for “not turning to community roles” are framed in terms of certainty.

- *“because they feared the strong sedation that invariably came, either directly or indirectly”*

The example illustrates a “closed” scenario where sedation is viewed as a certain outcome, leaving no room for the anticipations of other results after interacting with community roles. The use of this language modality limits the narrative trajectory a person can create: it pre-defines potential discourses about and from the listener in present and anticipatory terms.

**Table 9:** Processual analysis results: using roles to manage critical voice implications

	Hearers	Professionals	Family and friends
**Discursive Repertories**	Certify Reality: 32.26%	Certify Reality: 41.18%	Evaluation: 40%
	Description: 12.9%	Specification: 17.65%	Specification: 20%
	Generalization: 9.68%	Description: 11.76%	Cause: 20%
**Dialogic Weight**	3.9 dW	2.7 dW	2.5 dW
**Arcipelagos of Meaning**	Management issues	Management with Private	Management with Public Institutions
	Management with Association	Management with Public Institutions	Management issues
	Relatives	Support	Hospital wards

Interactively, this has critical repercussions not just for those who might miss out on support options, but also for the roles responsible for providing these services. They might have to manage these personal beliefs presented by different users. Regarding the role of “Hearers,” they tend to use generative modes more frequently to convey reasons for not engaging with community roles. Consider the example:

- *“I can’t afford it economically, and I’m afraid of finding out that I would need to be helped with psychotropic drugs, therapies, or even confinement.”*

This language use modality is based on common criteria, enabling the audience to engage with the content and propose potential strategies for managing these critical aspects. By using these DRs, in fact, interlocutors can position themselves as active community members, thus contributing to the management process of voice-related implications. This example shows how critical issues can be conveyed for generative use: not to maintain the status quo, but as a starting point for creating an alternative reality, thereby countering stereotypes and stigma.

## Discussion

### Discussion of the first research area results

The results in First research area: voice description section showed how respondents commonly rely on rhetorics incorporating personal references and absolutist perspectives that frame voice-hearing as a factual reality, resulting in its stereotyping and stigmatization.

This approach is mainly adopted by groups identified as “Externals” and “Family and Friends”, indicating a community tendency to stereotype voice-hearing based on these groups’ personal theories. At the same time, Voice Hearers and Professionals consistently use discursive modalities that disrupt stereotypical perceptions of voice-hearing, potentially contributing to the improvement of people’s health.

On an operational level, thus, the involvement of Voice Hearers and Professionals in sharing diverse perspectives can play a crucial role in addressing stereotypes about voice-hearing in the community [5, 7]. Effective interventions should focus on discussing various aspects of voice-hearing without imposing explanations or connotations, thereby minimizing conflicts with participants’ pre-existing beliefs or expectations. Utilizing neutral and relatable discourse elements enhances the chances of participants valuing the shared information, which encourages them to contribute with their own viewpoints [21].

In this regard, Voice onset section showed us how voice-hearing typically occurs at a young age (see Fig. 4) and it is negatively labeled by professionals and Family and Friends. Health promotion efforts should focus on raising awareness in community hubs frequented by this age group: schools, universities, youth centers.

Moreover, Voice Hearers reports are a valuable resource for preempting potential scenarios triggered by voice-hearing events. By adopting a proactive (ex-ante) approach, texts like the excerpts offered in Voice onset section could be instrumentally used to develop skills to manage the phenomenon, as well as anticipating critical elements that could arise.

Focusing specifically on the stigma issue, we observed a community’s tendency to portray voice-hearing through Stabilization DRs. In this regard, Dialogic Science allows us to anticipate how, instead of directly challenging the community’s negative portrayal of voice-hearing, it would be more effective to change the “discursive modality”.

For example, rather than disputing voices as positive or negative, encourage descriptions of personal experiences with voice-hearing. For instance, asking *“Could you describe an episode where the voices were threatening?”* allows for open expression and understanding of the reasons behind negative connotations. This approach, emphasizing relatable descriptions, aims to shift the argumentative style and foster diverse management strategies and contributions from various community roles.

### Discussion of the second research area results

Second research area: voice implications section showed us how, across the four study groups, there’s a common trend of attributing a predominantly negative impact to voice-hearing. This narrative contributes to stereotypes about voice-hearing and has critical consequences for voice hearers and their close associates.

At the same time, about 30% of the Voice Hearers group perceives the impact of voice-hearing on their lives as medium to low. Moreover, both Voice Hearers and Professionals frequently use Generative DRs. Despite being linked to the critical aspects of voice-hearing, the relatable and recognizable nature of these narratives allows for a deeper exploration of related issues, facilitating the development of management strategies with multiple community roles.

Like for the previous area, interventions tackling the stigma issue should not be focused only on changing the connotation of voice-hearing from negative to positive, as they might lead to controversy between the community’s personal theories and the proposed intervention; but rather on how these issues are reported.

Referring to Dialogic Science references, interventions should aim at transforming absolute narratives to ones that allow interlocutors to share the criteria behind their perception of voice-hearing. This can be achieved through strategic questions like *“What aspects of your life has the voice impacted?”* or *“Could you describe an episode where the voice had an impact on [specific aspect]?”*. Both questions facilitate a detailed exploration of the elements underpinning the perception of voice-hearing as critical, hence changing the discursive modality.

### Discussion of the third research area results

The results described in Third research area: voice management strategies section highlighted a consistent trend: the use of DRs focusing on maintaining existing management strategies, thereby limiting the exploration of new scenarios. This approach often frames voice-hearing as an inherently critical element to be managed, influencing individuals’ life paths and potentially leading to problems when unanticipated scenarios arise.

Concurrently, data reveal a widespread presence of self-management techniques across these roles, indicating the availability of resources and capabilities, either informally developed or formally acquired. These skills suggest the possibility of moving beyond traditional community roles, which are typically seen as passive recipients of institutional services, towards a more active and collaborative approach in managing the complexities of voice-hearing.

The operational strategies for managing voice-hearing could involve two interconnected approaches. The first is to challenge the perception of voice-hearing as unmanageable by altering the discursive processes. This involves moving beyond simply providing knowledge about managing critical aspects and instead, encouraging the use of alternative discursive modalities to clarify the criteria behind the perceived unmanageability.

By actively involving the recipient in the management process, this approach promotes descriptive and anticipatory thinking for alternative outcomes. Concurrently, the second approach focuses on the self-management methods developed by voice hearers. This strategy encourages the description of these methods using relatable elements, moving away from personal theories to enhance the recognisability of the discourse.

By combining these narratives with texts that utilize generative language modalities, guidelines and shared practices can be developed. These would be valuable in training courses for various community roles, both professional and non-professional, who interact with voice-hearing, thus leveraging experiential knowledge for the community’s broader benefit.

### Discussion of the fourth research area results

Fourth research area: interactions with community roles section showed how, in managing voice-hearing, there’s a tendency across respondent groups to view interactions with community roles in absolute and personal terms, reflecting a reliance on individual theories about the roles’ functions. This approach risks causing controversies between personal beliefs and the actual roles of service providers.

Additionally, community services are often sought after critical situations have already emerged, as shown in Fig. 15a and b. This reactive, post-hoc engagement with services hinders the effectiveness of proactive, ex-ante health promotion efforts. At the same time some respondents used Generative DRs to argument their choice for not seeking help from community roles, providing further insights for analyzing the needs of AVHs hearers (Not using roles to manage critical voice implications section).

Additionally, many respondents, especially Voice Hearers, have independently developed skills to manage voice-hearing, offering a valuable resource for improving and implementing related training programs. In light of this, managing AVHs should involve two key aspects.

Firstly, addressing the critical relationship dynamics between community members and health services. Secondly, responding to the need for alternative, non-institutional intervention methods, as exemplified by the HVM.

However, over-reliance on these associations could still lead to a delegation of responsibilities, risking to fall again in an emergency-focused management. To counteract this, a collective responsibility approach is needed, where all community roles actively participate in redefining narratives around critical conditions, ensuring a balanced and inclusive management of voice-hearing [23].

## Conclusion

This study was born from the need to address the pressing public concerns surrounding AVHs stigmatization. In accordance with the sociolinguistic literature [17, 18], we conceptualized AVHs stigma as a social process, interactively constructed and subject to ongoing refinement and reevaluation; thus, always open to change [34].

According to this perspective, each member of the community potentially contributes to the shaping of this construct, thereby influencing the nature of these interactive outcomes. Starting from this we navigated beyond traditional strictly cognitive and biomedical perspectives, highlighting how language plays a pivotal role in shaping, challenging, and perpetuating the discourses about AVHs.

We collected the narratives on AVHs of 278 respondents on a national level and analyzed them in terms of their interactive pragmatic implications. To do so we referred and presented an innovative textual analysis approach: Dialogic Science and MADIT, which have been instrumental in revealing the shape of the narratives building AVHs’ stigma.

One of the potential contributions of this study lies in the insights gleaned through our methodology, which extends beyond content analysis of the collected narratives (what is said), allowing to observe how a topic is configured. The main findings revolve around two main areas. (1) Respondents tend to define AVHs in absolute and stereotypical terms, limiting narrative diversity. This approach, often stigmatizing, solidifies AVHs as a fixed element in a person’s biography. (2) Local service narratives suggest a delegation to institutional roles as the sole managers of AVHs, dismissing the potential contributions of other community roles.

This perspective restricts active participation in managing AVH-related issues and exposes the risk of increased stigmatization. At the same time, the study revealed a range of discursive productions that offer a contrasting perspective to the previously described processes, potentially facilitating change, especially from the group of the “Hearers”. These discourses, characterized by the use of shareable and possibilistic language use modalities, provide valuable material to challenge the absolutist and personal theories surrounding AVHs.

Drawing from these narratives, in the Discussion section we used Dialogic Science references to provide operational insights and suggestions instrumental for the creation of interventions aimed at tackling stigma and supporting awareness towards AVHs. The examples demonstrate how adopting a processual perspective enables the formulation of strategic questions that address the interactive mechanisms underlying stigma. This approach promotes the generation of diverse narratives, integrating multiple community roles in addressing stigma through a comprehensive societal lens, thereby fostering social cohesion.

In this regard, it is emphasized that the measure provided by Dialogic Science could be effectively applied to assess and compare the outcomes of these interventions, offering a solution to the challenges associated with evaluating their effectiveness [13].

The research here described comes with some limitations: the current lack of validation for our ad-hoc built questionnaire and the sole focus on an Italian sample. Another limitation is the recruitment of Hearers and Relatives primarily through “Associazione Nazionale Sentire le Voci”. This may have resulted in a sample who are more accustomed to discussing their experiences, potentially influencing the narrative patterns observed in the study. In the future, hence, we aim to expand the number of Italian participants in order to conduct a first national-level validation of the tool, leveraging both the partnership with “Associazione Nazionale Sentire le Voci” and with other institutions. Then, given the applicative transversality of the tool (deriving from the assumptions of Dialogic Science and MADIT), we aspire to validate it also at an international level with different languages.

Finally, we note that recent sociological research has increasingly attended to the macro-level dimensions of stigma, shedding light on its structural causes, population-level consequences, and collective responses [35, 36]. However, as Clair [37] emphasizes, there is a critical need to bridge micro- and macro-level analyses to understand how stigma’s causes and consequences vary across different social groups. For example, Farrimond [34], adopting a structural macro-perspective, accentuates stigma’s processual nature, illustrating how stigma’s strength can be either reinforced or mitigated by the discursive interactions among social actors. In light of this, quantitatively assessing texts contribution to stigma, Dialogic Science’s dW could be used to analyze the interactions within networks of social actors, gauging how they collectively contribute to either exacerbating or alleviating stigma.

## Data Availability

The data that support the findings of this study are available on request from the corresponding author [DB]. The data are not publicly available since they contain information that could compromise research participant privacy and consent.

## Appendix A: Discursive Repertories - Glossary

**Table:**

Modality	Description
**I level**	
**Certify Reality - CR (Stabilisation)**	Discursive modality that configures reality by stating a clear, certain and unalterable state of things. The possibility of transformation is unforeseen for this reality.
**Description - DS (Generative)**	Discursive modality that configures reality as a common heritage that does not belong exclusively to any narrator and it needs everyone’s contribution to be maintained. It configures a current or past reality as if the narrator were responding to a question starting with “how” instead of “why”.
**II level**	
**Specification - SI (Hybrid)**	Discursive modality that configures reality by providing a generation or Stabilisation of an explicit and detailed description regarding the configuration it is associated with, limiting its range of application to what is expressed.
**Possibility - PS (Hybrid)**	Discursive modality that configures reality by using one’s own and exclusive criteria as the only argumentative foundation, without making them explicit and describing them in order to make them shared. It configures reality in probabilistic, possibilistic and uncertain terms.
**III level**	
**Opinion - OI (Stabilisation)**	Discursive modality that configures reality by making explicit that the contents are valid and delimited within the narrator’s own and exclusive perspective.
**Targeting - TG (Generative)**	Discursive modality that configures reality in order to set an objective/purpose/goal to another part of the text, defining actions, strategies, interventions, etc. Enables the triggering of a discursive configuration aimed at the pursuit of the defined objective/purpose/goal and, in this way, generating modalities belonging to the generative class and of maximum generative impact.
**Cause of Action - CA (Stabilization)**	Discursive modality that configures reality through empirical-factual connections of cause-effects with value of truth, which determine an immutable course of events. The argumentation is not epistemologically founded.
**Confirmation - CP (Hybrid)**	Discursive modality that configures reality by validating and supporting what expressed through the Repertory to which it relates.
**IV level**	
**Contraposition - CT (Stabilisation)**	Discursive modality that configures reality through parallelism between two or more discourse’s parts, which are connected in terms that one excludes the other. The criteria that allow exclusion are not made explicit.
**Implication - IP (Hybrid)**	Discursive modality that configures reality shaping the narrator’s own and exclusive position regarding probable situations that could occur and that have not yet occurred, through a cause-effect rhetorical argumentative link. Those situations are reported in a tense (and time) following the one related to the main action (present perfect-simple past or present or future, present-future, etc.).
**Judgement - JM (Stabilisation)**	Discursive modality that configures reality according to CR’s processual properties by using moral and/or qualitative attributes without making explicit the criteria used, shaping the narrator’s own and exclusive reality which therefore is not shareable.
**Prediction - PV (Stabilisation)**	Discursive modality that configures realities defining/stating a future scenario as a certain result of the development of a current scenario through a cause-effect rhetorical argumentative link.
**Justification - JT (Stabilisation)**	Discursive modality that configures reality by entailing Stabilisation of the “current state of things”: it associates a situation to a previous one in order to legitimize a “state of things”, obstructing the use of other ways to handle or change what is happening.
**Non-Answer - NA (Stabilisation)**	Discursive modality that configures reality in order to avoid the asked question - according to CR’s processual properties - establishing a “state of things” in which the narrator does not adhere properly to the process introduced by the question itself.
**Comment - CM (Stabilisation)**	Discursive modality that configures reality in an inappropriate and irrelevant way to what is asked in the question following the narrator’s own and exclusive criteria, which are neither made explicit nor sharable. The argumentation does not allow to answer the question asked and it uses CR’s processual properties.
**Generalization - GE (Stabilisation)**	Discursive modality that configures reality by responding inadequately to the question asked and using cross-context argumentations, thus not covering what is required. The criteria used are not epistemologically founded.
**Evaluation - EU (Hybrid)**	Discursive modality that configures reality by stating a “state of things” funded on the narrator’s own and exclusive criteria, which, although explicit, are non-sharable.
**Declaration of Aims - DA (Hybrid)**	Discursive modality that configures reality by transposing the object of the request in a future perspective, without elements of certainty and probability as foundation.
**Proposal - PP (Generative)**	Discursive modality that configures uncertain reality, possible in an achievable way and aimed at handling what is requested/offered according to TG’s processual properties.
**Delegating to others - DE (Stabilisation)**	Discursive mode that configures reality by delegating to third parties processes that are proper and exclusive to the narrator.
**V level**	
**Prescription - PT (Hybrid)**	Discursive modality that configures reality as orders/directions given by a third “point of view” position compared to the narrator’s one. Establishes rules and/or objectives and/or roles to follow, in terms of what one “has to do” or “has not to do”. The argumentation acquires a structure founded on a relation of necessity set by a part of the text.
**Reshaping - RS (Hybrid)**	Discursive modality that configures realities that limit the generative potential of what the configuration offers. The argumentation’s reference is third and not referable to the narrator.
**Consideration - CS (Generative)**	Discursive modality that configures reality by proposing an argument which uses criteria of analysis that can be shared among several interlocutors, namely that do not belong to any narrators exclusively, but need all of their contribution to maintain them (the criteria).
**VI level**	
**Anticipation - AT (Generative)**	Discursive modality that configures reality through an argumentation shaped according to CS’s processual properties. This Repertory configures many different and uncertain situations that can occur and that have not yet occurred using PS’s processual properties.

## Appendix B: Questionnaire

Below we present the protocol of questions administered to the “Hearers” group. We highlight how the other questionnaires followed the same structure. However, due to the specificity of the interviewed roles, the questions have been adapted both on a grammatical level (i.e. transposing the questions from the second to the third person) and eliminating the questions which it was not possible to answer by virtue of the role held.

- Area 1: Describe the discursive configuration regarding the voices and episodes in
  - 1a) How many voices do you hear?
  - 1b) How old were you when you started hearing the voices?
  - 1c) How would you describe the first time you heard the voices? (describe where you were, how the voice manifested, what happened, etc.)
  - 1d) Do you think the voices you hear originated in one or more specific moments? [Yes] [No]
  - 1e) If yes, how would you describe that moment?
  - 2a) How would you describe the voices you hear?
  - 2b) Where do the voices you hear at that moment come from? (internal to you, coming from outside, in which part of the body or places where you are, etc.)
  - 2c) What do the voices tell you? (also report conversations/sounds/etc.)
  - 3a) The voices you hear are: [positive][negative][neutral]
  - 3b) Are the people you know (friends, family, etc.) aware that you hear voices? [Yes][No]
  - 3b1) If yes: How do the people you know (friends, family, etc.) describe these voices you hear?
  - 3b2) If no: If you were to tell the people you know (friends, family, etc.) that you hear voices, what would these people say about the voices you hear?
- Area 2: Describe the discursive configuration related to the implications and challenges of hearing voices.
  - 4a) Which aspects of your life are most affected by the voices (e.g., daily life, school, work, habits, relationships, hobbies, etc.)?
  - 4b) Using a scale from 1 to 7, how much do the voices you hear affect these aspects of your life (where 7 is a significant influence and 1 is minimal)? [1-7]
  - 4c) Why did you indicate that value?
  - 5) How would you describe yourself in the moments when you hear the voices?
  - 6) Have you ever been hospitalized? [Yes][No]
  - 6a) If yes: Could you describe the situation that led to your hospitalization? (so the moment before being hospitalized)
  - 7) Could you describe a situation you have experienced, where the voices have influenced aspects of your life, referred to in question 4a?
- Area 3: Describe the discursive configuration regarding the management of the implications and challenges of hearing voices.
  - 8) How did you deal with that situation?
  - 9) Going back to the aspects of your life (that you entered in question 4a) that are most affected by the voices; how would you describe what you do to deal with these situations?
  - 10) Are you currently taking psychotropic medicament? [Yes][No]
  - 10a) Which medicament are you taking?
  - 10b) In what quantity?
- Area 4: Describe the configuration of roles/services used in managing the implications and challenges of hearing voices.
  - 11) To manage the aspects of your life where you hear voices, have you used local services to handle such situations?
  - 11a) If yes: which services did you turn to?
  - 12) Did you turn to someone to handle difficult situations you found yourself in?
  - 12a) If yes: whom did you turn to? [Association][Psychosocial Center][Mental Health Center][Psychiatrist][Psychologist][Residential Facility]
  - 12b) Why did you turn to the roles you indicated? Explain why for each role.
  - 12c) If no: Why didn’t you turn to anyone?
  - 12d) Who would you turn to now?
  - 12e) Why would you turn specifically to the roles you mentioned in question 11d?
  - 13) In what way and how did this role(s) you indicated help you? Explain for each role how and in what they have helped you (if there are multiple roles)
  - 14) What kind of support do you think would be useful to receive when you hear voices?

## Appendix C: Arcipelagos of Meaning

**Table:**

**Arcipelagos IT**	**Arcipelagos ENG**
Luogo Pubblico	Public Place
Luogo Privato	Private Place
Scuola	School
Lavoro	Work
Reparti Ospedalieri	Hospital Wards
Esperienza da solo	Experience Alone
Esperienza con altri	Experience with Others
Parenti	Relatives
Colleghi	Colleagues
Amici	Friends
Partner	Partner
Docenti	Teachers
Professionisti	Professionals (psychiatrists/psychologists)
Genesi del fenomeno	Genesis of the Phenomenon
Genesi traumatica	Traumatic Genesis
Genesi non-traumatica	Non-Traumatic Genesis
Interazione in cui gli altri esercitano un ruolo “attivo”	Interaction in Which Others Play an “Active” Role
Interazione in cui gli altri esercitano un ruolo “passivo”	Interaction in Which Others Play a “Passive” Role
Bullismo/Derisione	Bullying/Mockery
Interazione con altri connotata come stressante	Interaction with Others Deemed Stressful
Identità della voce definita	Defined Voice Identity
Entità Spirituali	Spiritual Entities (e.g., Satan)
Defunti	Deceased
Persone Conosciute	Acquaintances
Identità della voce indefinita	Undefined Voice Identity
Voce Maschile	Male Voice
Voce Femminile	Female Voice
Voce interna	Inner Voice
Voce esterna	External Voice
Voce molteplice	Multiple Voices
Voci giudicanti	Judging Voices
Voci giudicanti positive	Positively Judging Voices
Voci giudicanti negative	Negatively Judging Voices
Voci prescrittive	Prescriptive Voices
Voci informative	Informative Voices
Voce come reale	Voice as Reality
Voci supportive	Supportive Voices
Contesto d’influenza della voce specifico	Specific Voice Influence Context
Ambito lavorativo	Work Environment
Ambito scolastico	School Environment
Ambito relazionale	Relational Environment
Contesto d’influenza della voce generale	General Voice Influence Context
Voce influente	Influential Voice
Voce ininfluente	Inconsequential Voice
Effetto negativo della voce	Negative Effect of the Voice
Effetto di paura della voce	Fear Effect of the Voice
Effetto limitante della voce	Limiting Effect of the Voice
Effetto di curiosità rispetto alle voci stesse	Effect of Curiosity Regarding the Voices Themselves
Effetto della voce di distacco dalla realtà	Effect of the Voice Detaching from Reality
Effetto positivo della voce	Positive Effect of the Voice
Autogestione	Self-Management
Gestione con Psicologo	Management with Private Psychologist
Gestione con Associazione	Management with Association
Gestione con Professionista	Management with Private
Gestione con Psichiatra	Management with Private Psychiatrist
Gestione con Parenti	Management with Relatives
Gestione con Amici	Management with Friends
Gestione con uditore/i	Management with Listeners
Gestione tramite Istituzioni Sanitarie Pubbliche	Management with Public Health Institutions
Gestione tramite Comunità/Strutture Residenziali	Management through Community/Residential Facilities
Criticità nella Gestione	Management Issues
Impossibilità (o Incapacità) di gestione	Inability (or Incapacity) to Manage
Suicidio	Suicide
Strategie di Gestione	Management Strategies
Trattamento Farmacologico	Pharmacological Treatment
Ascolto/Comprensione da parte di altri	Listening/Understanding by Others
Informazioni/Formazione	Information/Training
Supporto	Support
Persona che deve affrontare difficoltà	Person Facing Difficulties
Persona che deve affrontare un trauma	Person Facing a Trauma
Persona che deve affrontare una psicopatologia	Person Facing a Psychopathology
Persona che deve gestire una carico emotivo	Person Managing an Emotional Burden
Normalità	Normality
Persona normale che sente le voci	Normal Person Hearing Voices
Sintomatologia fisica	Physical Symptoms
Paranormale	Paranormal
Nameless	Nameless
Altro	Other

## Footnotes

1 https://www.sentirelevoci.it/

2 Goffman [30], in fact, defining stigma stresses “*The term stigma, then, will be used to refer to an attribute that is deeply discrediting, but it should be seen that a language of relationships, not attributes, is really needed*” ([30], p. 3). As Tomar and Thornicroft [31] notes, the second part of the definition underscores the interactive dimension of stigma, showing how a contents acquires its “stigmatising value” only through a web of interactions.
